# Abstract

**DOI:** 10.1002/jcsm.12551

**Published:** 2020-02-12

**Authors:** 


**1-16**



**Cancer cachexia induces iBAT thermogenesis in UCP1^−/−^ mice**


Magno A. Lopes^1^, Jessica S. Santos^1^, Marcela J.O.A. Carreira^1^, Luis F.G. Valdivia^1^, Luana G. Leal^1^, Sydney B. Peres^2^ and **Miguel L. Batista Jr**
^1^



^1^
*Laboratory of Adipose Tissue Biology, Integrated Group of Biotechnology, University of Mogi das Cruzes, Mogi das Cruzes, Brazil;*
^2^
*Department of Physiological Sciences, State University of Maringá, Maringá, Brazil*



**Background:** Cancer cachexia (CC) is a multifactorial syndrome with an unknown aetiology. The main symptom is the progressive body weight loss. During the development of the syndrome, there is an increase in uncoupling protein 1 (UCP1) in white adipose tissue (WAT) and intrascapular brown adipose tissue (iBAT), resulting in increased energy expenditure and heat generation. Recently, it was proposed that energy expenditure in CC does not depend on UCP1 but on an independent thermogenic pathway. In this sense, this study aimed to evaluate the thermogenic activity of UCP1 knockout mice during CC.


**Methods:** Male C57BL/6 mice (8–10 week old) wild‐type (WT) and knockout for UCP1 (UCP1^−/−^) were subcutaneously inoculated with 200 μL (3.5 × 10^5^) of Lewis Lung Carcinoma Cell line (TB) and vehicle saline (CO). After 28 days, animals were submitted to thermal infrared measurements (T460, emissivity of 0.98, FLiR Systems). After analysing, animals were sacrificed, and the WAT subcutaneous (SC) and epididymal (EP), BAT, and gastrocnemius muscle (GA) were collected.


**Results:** UCP1^‐/‐^ TB presented higher maximum body temperature (10.4‐fold, *P* = 0.0500) and tendency to increase the maximum temperature of BAT (*P* = 0.0750) when compared to UCP1^‐/‐^ CO. In addition, UCP1^‐/‐^ TB have preserved both the body weight loss (11.0‐fold, *P* = 0.0101) and the values of the index of cachexia (45.4%, *P* = 0.0305) when compared to TB group. The adipose tissue atrophy (EP, 14.6‐fold and BAT, 15.3‐fold) was also attenuated in UCP1^‐/‐^ TB group when compared to TB group.


**Conclusions:** In general, the results showed that UCP1^‐/‐^ animals presented attenuation in the main markers of CC. In addition, the BAT of these animals showed an increase in their thermogenic capacity, suggesting that its activation, at least in part, may be activated by an UCP1‐independent pathway.


**1-17**



**Lack of synergy between β‐agonist treatment and a blockage of sarcoplasmic calcium flow in a rat cancer cachexia model**



**Queralt Jové**
^1^, Sílvia Busquets^1,2^, Marta Castillejo^1^, Baptiste Jude^3^, Patricia Mejías^1^, Francisco J. López‐Soriano^1,2^ and Josep M. Argilés^1,2^



^1^
*Cancer Research Group, Departament de Bioquímica i Biomedicina Molecular, Facultat de Biologia, Universitat de Barcelona, Barcelona, Spain;*
^2^
*Institut de Biomedicina de la Universitat de Barcelona, Barcelona, Spain;*
^3^
*Laboratoire de Physiologie, ORPHY, IBSAM, Université de Bretagne Occidentale, Brest, France*



**Background:** Accelerated muscle and adipose tissue loss are two of the main aspects of cancer cachexia. The use of β2‐agonists, formoterol in particular, has proven to be very successful in the treatment of the syndrome in preclinical models. The aim if the present investigation was to study the effects on body weight loss in tumour‐bearing animals of a combination of formoterol and dantrolene, an inhibitor of the ryanodine receptor 1 (RyR1) involved in the sarcoplasmic reticulum calcium flow.


**Methods:** Rats were divided into two groups, namely controls (C) and tumour‐bearing (TB). TB group was further divided into four subgroups: untreated (saline as a vehicle), treated with formoterol (TF) (0.3 mg/kg body weight in saline, subcutaneous (s.c.), daily), treated with dantrolene (TD) (5 mg/kg body weight in saline, subcutaneous (s.c.), daily), and double‐treated treated (TFD) with formoterol (0.3 mg/kg body weight, subcutaneous (s.c.), daily) and dantrolene (5 mg/kg body weight, subcutaneous (s.c.), daily). Seven days after tumour transplantation, muscle weights, grip force, and total physical activity were determined in all experimental groups.


**Results:** While formoterol has, as in previous studies, a very positive effect in reducing muscle weight loss, dantrolene had no effects, neither on skeletal muscle or any of the parameters studied. Finally, the combined treatment (formoterol and dantrolene) did not result in any significant benefit on the action of the β2‐agonist.


**Conclusions:** It is concluded that in the preclinical cachectic model used, no synergy exists between β2‐agonist treatment and the blockade of sarcoplasmic calcium flow.


**1-18**



**Chronic kidney disease induces muscle wasting via soluble pro‐cachectic factors**



**Francesca Solagna**
^1^, Robert Mitchell^2^, Charlotte Mayer^3^, Saleh Omairi^4^, Antonios Matsakas^5^, Olli Ritvos^6^, Arja Pasternak^7^, E. Hoxha^1^, Temel Kilic^3^, Andrea Paolini^2^, Nicola Wanner^1^, Jan‐Eric Turner^1^, Julian Schulze zur Wiesch^8^, Maja Lindenmeyer^1^, Clemens D. Cohen^9^, Oliver Kretz^1,4^, Victor G. Puelles^1,10^, Ketan Patel^3,7^ and Tobias B. Huber^1^



^1^
*III. Department of Medicine, University Medical Center Hamburg‐Eppendorf, Hamburg, Germany;*
^2^
*School of Biological Sciences, University of Reading, Reading, UK;*
^3^
*Department of Neuroanatomy, Institute of Anatomy, Medical Faculty, University Freiburg, Freiburg, Germany;*
^4^
*Department of Medicine, Renal Division, University Medical Centre Freiburg, Freiburg, Germany;*
^5^
*Molecular Physiology Laboratory, Centre for Atherothrombosis and Metabolic Disease, Hull York Medical School, Hull, UK;*
^6^
*Department of Bacteriology and Immunology, University of Helsinki, Helsinki, Finland;*
^7^
*Freiburg Institute for Advanced Studies and Center for Biological System Analysis, Freiburg, Germany;*
^8^
*I. Department of Medicine, University Medical Center Hamburg‐Eppendorf, Hamburg, Germany;*
^9^
*Nephrological Center, Medical Clinic and Policlinic IV, University of Munich, Munich, Germany;*
^10^
*Department of Nephrology and Centre of Inflammatory Diseases, Monash University, Melbourne, Australia*


Chronic kidney disease (CKD) represents the progressive and permanent loss of kidney function. While muscle wasting is a prominent feature of CKD and significantly increases morbidity and mortality, this interorgan signalling network remains poorly understood. Here, we identified muscle wasting in a mouse model of CKD (KIf3a deficiency), where we discovered an increased renal production of soluble pro‐cachectic factors (i.e. Activin A) as shown by both transcriptional regulation and increases in plasma levels, an observation that was also confirmed in patients at different stages of CKD. Furthermore, pharmacological blockade of the identified pro‐cachectic factor in mice prevented muscle wasting and progression to CKD by reducing its levels in the plasma—both a direct neutralization and recovery of kidney function. Together, this study uncovers a previously unrecognized crosstalk between kidney and muscle and provides a potential therapeutic strategy for muscle wasting in patients with CKD.


**2-09**



**Interleukin‐6 controls microRNA‐regulated networks in skeletal muscle cells**


Paula P. Freire^1,2^, Jianming Liu^2^, Sarah S. Cury^1^, Letícia Oliveira^1^, Grasieli de Oliveira^1^, Diogo de Moraes^1^, Geysson J. Fernandez^1,3^, Maeli Dal‐Pai‐Silva^1^, Da‐Zhi Wang^2,4^ and **Robson F. Carvalho**
^1^



^1^
*Department of Morphology, Institute of Biosciences, São Paulo State University, UNESP, Botucatu, Brazil;*
^2^
*Department of Cardiology, Boston Children's Hospital, Harvard Medical School, Boston, MA, USA;*
^3^
*Faculty of Medicine, University of Antioquia, UdeA, Medellín, Colombia;*
^4^
*Harvard Stem Cell Institute, Harvard University, Cambridge, MA, USA*


Systemic inflammation contributes to the development of cachexia, and the pro‐inflammatory cytokine interleukin‐6 (IL‐6) has emerged as a critical factor related to muscle wasting during disease. Regulation of gene expression by microRNAs (miRNAs) in skeletal muscles integrates regulatory networks, which are predicted to involve thousands of transcripts through different mechanisms. Our objective was to analyse the global miRNA expression profile of skeletal muscle cells atrophy induced by IL‐6 to uncover potential miRNAs involved with this catabolic condition. Genome‐wide gene expression profiles of miRNAs were performed by using TaqMan Low‐Density Arrays in C2C12 myotubes treated with IL‐6, followed by in silico predictions for miRNA targets. High concentrations of IL‐6 induced myotube atrophy and decreased the levels of *Myh7* and *e‐MHC*. Moreover, we identified 20 differentially expressed miRNAs in C2C12 myotubes in response to IL‐6 (five upregulated, and 15 downregulated). Gene Ontology analysis of the predicted targets of these miRNAs revealed potential posttranscriptional regulation of genes involved in cell differentiation, apoptosis, migration, and catabolic processes. Interestingly, the miR‐497 was suppressed by the treatment with IL‐6, and it was found in the literature to be downregulated in other muscle catabolic conditions. Thus, we used miR‐497 mimics and inhibitors to explore the function of this miRNA. The miR‐497 changed cell cycle target genes, such as *Ccnd2* and *Ccne1*, but did not alter myoblast proliferation, as assessed by the EdU assay. Also, miR‐497 mimic induced myotubes atrophy. The miR‐497 inhibitor did not change myotubes diameters but resulted in overexpression of the miR‐497 target genes *Insr* and *Igf1r*. These genes are involved with the insulin‐like growth factor pathway and are overexpressed in muscle samples of cachexia models (GSE48363, GSE63032, GSE24111, and GSE51931). Our miRNA analysis identified miRNA‐regulated networks and suggests that miR‐497 is involved in a compensatory mechanism to muscle atrophy in response to IL‐6.

This study was financed in part by the Coordenação de Aperfeiçoamento de Pessoal de Nível Superior—Brasil (CAPES)—Finance Code 001 (CAPES‐PrInt‐UNESP) and by the Grant 12/13961‐6, São Paulo Research Foundation (FAPESP/Brasil).


**2-11**



**Transcriptomic analysis reveals heme‐related genes are downregulated in chronic obstructive pulmonary disease cachexia**



**Ava C. Wilson**
^1,2^, Preeti L. Kumar^2^, S. Lee^3^, Margaret M. Parker^3^, Itika Arora^2^, Jarrett D. Morrow^3^, Emiel F.M. Wouters^4^, Richard Casaburi^5^, Stephen I. Rennard^6,7^, David A. Lomas^8^, Alvar Agusti^10^, Ruth Tal‐Singer^11^, Mark T. Dransfield^2^, Surya P. Bhatt^2^, Victor J. Thannickal^2^, Hemant K. Tiwari^12^, Craig P. Hersh^3,9^, Peter J. Castaldi^3,9^, Edwin K. Silverman^3,9^ and Merry‐Lynn N. McDonald^1,2,13^



^1^
*Department of Epidemiology, School of Public Health, University of Alabama at Birmingham, Birmingham, AL, USA;*
^2^
*Division of Pulmonary, Allergy and Critical Care Medicine, Department of Medicine, University of Alabama at Birmingham, Birmingham, AL, USA;*
^3^
*Channing Division of Network Medicine, Brigham and Women's Hospital, Harvard Medical School, Boston, MA, USA;*
^4^
*Centre of expertise for chronic organ failure, Horn, The Netherlands;*
^5^
*Rehabilitation Clinical Trials Center, Los Angeles Biomedical Research Institute at Harbor‐UCLA Medical Center, Torrance, CA, USA;*
^6^
*Department of Medicine, Nebraska Medical Center, Omaha, Nebraska, USA;*
^7^
*BioPharmaceuticals R&D, AstraZeneca, Cambridge, UK;*
^8^
*Division of Medicine, University College London, London, UK;*
^9^
*Division of Pulmonary and Critical Care Medicine, Brigham and Women's Hospital, Boston, MA, USA;*
^10^
*Fundació Investigació Sanitària Illes Balears (FISIB), Ciber Enfermedades Respiratorias (CIBERES), Barcelona, Catalunya, Spain Thorax Institute, Hospital Clinic, IDIBAPS, Univ. Barcelona, Barcelona, Spain;*
^11^
*GSK R&D, Collegeville, PA, USA;*
^12^
*Department of Biostatistics, School of Public Health, University of Alabama at Birmingham, Birmingham, AL, USA;*
^13^
*Department of Genetics, University of Alabama at Birmingham, Birmingham, AL, USA*



**Background:** Cachexia contributes to increased mortality and reduced quality of life in chronic obstructive pulmonary disease (COPD). COPD cachexia may be associated with underlying gene expression changes that could provide valuable insights for surveillance and drug development. Our goal was to identify differential gene expression signatures associated with COPD cachexia in current and former smokers.


**Methods:** We analysed gene expression data from a discovery cohort of COPD patients (COPDGene, *N* = 400) and assessed replication in ECLIPSE (*N* = 140). Whole blood expression data were generated using RNA‐sequencing in COPDGene and Affymetrix in ECLIPSE. In COPDGene, cachexia was defined as weight loss >5% in the past 12 months or low body mass index (BMI) and 1/3 criteria: decreased muscle strength (6 min walk distance), anaemia, and low fat‐free mass index (FFMI). In ECLIPSE*,* cachexia was defined as weight loss >5% in the past 12 months or low BMI and 3/5 criteria: low 6 min walk distance, anorexia, abnormal biochemistry (anaemia or high c‐reactive protein), fatigue, and low FFMI. Differential gene expression was performed using regression models comparing cachectic and noncachectic subjects, adjusting for confounders including age and sex. Gene set enrichment analysis was performed using MSigDB.


**Results:** Cachexia prevalence was 13.7% in COPDGene and 7.9% in ECLIPSE. In COPDGene, 23 genes were significantly differentially expressed (FDR‐*P* < 0.05) in cachectic versus noncachectic COPD patients. Replication analyses revealed 14/23 genes significantly replicated in ECLIPSE (*P* < 0.05) and were downregulated in each cohort. Several replicated genes are involved with heme metabolism (*ALAS2*, *ANK1*, *TNS1*, *SPTB*, *TRIM58*, *PPP2R5)* and biosynthesis (*ALAS2*, *SLC25A39*). Remaining significant genes (*ASCC2, CDC34, GUCD1, PLEK2, RILP, SMIM24, UBXN6*) are involved with DNA damage repair, protein metabolism, and ubiquitination.


**Conclusions:** Several genes were downregulated among cachectic COPD patients, including genes regulating heme metabolism. Impaired heme biosynthesis may contribute to cachexia through free‐iron buildup, oxidative tissue damage, and aberrant repair.


**2-12**



**Pancreatic tumour organoid conditioned medium negatively affects the smooth muscle cell contractile phenotype**



**Rianne D.W. Vaes**
^1^, Merel R. Aberle^1,2^, David P.J. van Dijk^1^, Annemarie A. van Bijnen^3^, Steven W.M. Olde Damink^1,3,4^ and Sander S. Rensen^1^



^1^
*Department of Surgery and NUTRIM School of Nutrition and Translational Research in Metabolism, Maastricht University, Maastricht, The Netherlands;*
^2^
*Department of Pharmacology and Toxicology and NUTRIM School of Nutrition and Translational Research in Metabolism, Maastricht University, The Netherlands;*
^3^
*Department of Surgery, Maastricht University Medical Centre, Maastricht, The Netherlands;*
^4^
*Department of General, Visceral and Transplantation Surgery, RWTH University Hospital Aachen, Aachen, Germany*



**Background:** Patients with pancreatic cancer often suffer from gastrointestinal symptoms which may be the consequence of underlying gastrointestinal motility problems. Although muscle loss in cachectic pancreatic cancer patients is most obvious in skeletal muscle, these clinical symptoms as well as our recent analysis of smooth muscle characteristics in cachectic patients suggest that cachexia manifests itself also in smooth muscle, a tissue responsible for contraction of the gastrointestinal tract. We aimed to investigate whether tumour cells from cachectic pancreatic cancer patients directly affect the smooth muscle cell (SMC) contractile phenotype.


**Methods:** 3D organoids were established from pancreatic tumour tissue of eight patients with a variable degree of cachexia. Human visceral SMCs were grown to confluency on basement membrane matrix coated surfaces under reduced serum conditions to induce a contractile phenotype and subsequently exposed to organoid conditioned medium (CM) (50% *v/v*). Markers of muscle atrophy, contractile machinery, and proliferation were evaluated by qPCR and Western blot. SMC proliferation and migration was also monitored by real‐time imaging.


**Results:** CM from pancreatic tumour organoids of cachectic patients did not affect expression of Atrogin‐1, a key E3‐ubiquitin ligase that is involved in skeletal muscle atrophy. Nevertheless, exposure to organoid CM caused reduced protein levels of α‐smooth muscle actin (α‐SMA) (1.4‐fold, *P* < 0.001) and smooth muscle protein 22‐α (SM22α) (2‐fold, *P* < 0.001), two key proteins involved in SMC contraction. Moreover, γ‐smooth muscle actin expression was significantly reduced (1.4‐fold, *P* < 0.001). Concurrently, expression of *S100A4*, a key protein involved in SMC proliferation, was increased (1.4‐fold, *P* < 0.001). In line, SMCs exposed to organoid CM showed a significantly reduced doubling time (control: 36.2 h vs. organoid CM: 29.9 h, *P* < 0.001).


**Conclusions:** Pancreatic tumour cells from cachectic patients secrete factors that diminish the contractile SMC phenotype, which may be the underlying cause of the frequently observed gastrointestinal motility problems in these patients.


**2-13**



***In vitro* chemotherapy response of pancreatic tumour organoids from cachectic and noncachectic patients**



**Merel R. Aberle**
^1,2^, Rianne D.W. Vaes^1^, Stefanie C. Hendrikx^2^, Jorne Ubachs^3^, Tessa T.J. Welbers^1^, Frederik‐Jan van Schooten^2^, Steven W.M. Olde Damink^1,4^ and Sander S. Rensen^1^



^1^
*Department of Surgery, NUTRIM School of Nutrition and Translational Research in Metabolism, Maastricht University, Maastricht, The Netherlands;*
^2^
*Department of Pharmacology and Toxicology, NUTRIM School of Nutrition and Translational Research in Metabolism, Maastricht University, The Netherlands;*
^3^
*Department of Obstetrics and Gynaecology, GROW School of Oncology and Developmental Biology, Maastricht University Medical Centre, Maastricht, The Netherlands;*
^4^
*Department of General, Visceral‐ and Transplantation Surgery, RWTH Aachen University, Aachen, Germany*



**Background:** Pancreatic cancer is a devastating disease with poor clinical outcome due to a lack of adequate systemic therapies. Additionally, 80% of patients suffer from cachexia, a syndrome of severe weight and/or muscle loss that is associated with reduced chemotherapy efficacy. Organoids are 3D cell cultures that can be used to investigate drug responses *in vitro*. This study aimed to identify differences in responses to chemotherapy in tumour organoids derived from cachectic versus noncachectic patients.


**Methods:** Organoids were established from resected tumour tissue from patients with pancreatic ductal adenocarcinoma (*n* = 9). Three cell lines (two from cachectic donors and one from a noncachectic donor) were exposed to a concentration range of four chemotherapeutics that are frequently used for the treatment of pancreatic cancer: gemcitabine, paclitaxel, irinotecan, and 5‐fluorouracil. After 5 days, cell viability was assessed using the CellTiter‐Glo® ATP assay.


**Results:** Significant differences were observed between the IC50 values of the three patients for gemcitabine and paclitaxel that indicate resistance to these drugs. This resistance was not restricted to the organoid lines coming from cachectic donors. For gemcitabine, one of the cachectic patients showed significant resistance (IC50 5.46 × 10^−2^ μM versus 6.43 × 10^−3^ μM and 9.09 × 10^−3^ μM, *P* < 0.005), whereas for paclitaxel, the noncachectic patient was significantly resistant (IC50 9.18 × 10^−3^ μM versus 2.22 × 10^−3^ μM and 1.92 × 10^−3^ μM, *P* < 0.01). For irinotecan and 5‐fluorouracil, the IC50 values were similar for all three patients.


**Conclusions:** Our present data show significant differences in chemotherapy sensitivity between organoid lines that are partly related to the cachexia status of the donor patient. Our aim is to relate the *in vitro* chemotherapy sensitivity to the cachexia status, patient treatment response, and survival. This can potentially lead to better selection of therapies for the treatment of cancer patients.


**2-14**



**ZIP14 as a mediator of cachexia in metastatic cancers**



**Anup Biswas**
^1^, Wanchao Ma^1^, Gang Wang^1,2^, Courtney Coker^1^ and Swarnali Acharyya^1,3^



^1^
*Institute for Cancer Genetics, Columbia University Medical Center, New York, NY, USA;*
^2^
*Weill Cornell Medical College, New York, NY, USA;*
^3^
*Herbert Irving Comprehensive Cancer Center, New York, NY, USA*


Category: Cachexia – Mechanisms, animal models

Metastasis contributes to the vast majority of cancer‐related deaths. Metastatic tumours secrete factors that systemically affect various organs leading to metabolic dysfunction and accelerated death. More than 80% of metastatic cancer patients experience a progressive and debilitating loss of muscle mass and function by a process known as cachexia. Cachectic patients suffer deterioration of cardiac and diaphragm muscles and often die prematurely due to respiratory and cardiac failure. The prognosis for these patients is further diminished by the fact that they are often too weak to tolerate standard doses of antineoplastic treatments. Cachexia is therefore an important determinant of therapeutic response, outcome, and patient survival in metastatic cancer patients. Although systemic metabolic derangements and sustained inflammation predominate in cachexia, the underlying molecular mechanisms driving its development are not well understood. Therefore, insights into the specific interventions that could treat cachexia are expected to improve treatment outcome, survival, and quality of life in cancer patients. We identified a metal ion transporter, ZIP14 that is upregulated in cachectic muscles from metastatic colon, lung, breast, and pancreatic cancer. We find that TNF‐α and TGF‐beta cytokines upregulate ZIP14 in muscles, which in turn increases the accumulation of intracellular zinc in muscle cells. Increased zinc influx in muscle cells degrades myosin heavy chain protein and blocks normal muscle differentiation. Germline ablation or muscle‐specific depletion of *Zip14* markedly inhibits cancer‐associated cachexia. Our study demonstrates a novel function of ZIP14 in muscle cells as a mediator of cachexia in metastatic cancers. Insights from this study can be used to develop therapeutic strategies to prevent cachexia and to improve the survival and quality of life in metastatic cancer patients.


**3-20**



**Effect of human cancer cachexia on the partition of lipids and inflammation in the liver**


Silvio Pires Gomes^1^, Daniela Caetano Goncalves^2^, Bruno Cogliati^3^, Ivanir Santana de Oliveira Pires^1^, Flávio Tokeshi^4^, Alcântara, Paulo Sérgio Alcantara^4^, José Pinhata Otoch^4,5^, Joanna Darck Carola Correia Lima^1^ and Estefania Simões^1^ and **Marilia Cerqueira Leite Seelaender**
^1,5^



^1^
*Cancer Metabolism Research Group, Institute of Biomedical Sciences, University of São Paulo, São Paulo, Brazil;*
^2^
*Federal University of São Paulo, UNIFESP, São Paulo, Brazil;*
^3^
*School of Veterinary Medicine and Animal Science, University of São Paulo, São Paulo, Brazil;*
^4^
*Universitary Hospital ‐ University of São Paulo, São Paulo, Brazil;*
^5^
*Department of Anesthesiology, Medical College of the University of São Paulo, São Paulo, Brazil*



**Background:** Cancer cachexia is a paraneoplastic wasting syndrome present in 50% of all patients and is up to 80% of those with advanced disease. Recent studies have suggested that the liver plays a key regulatory role in the pathogenesis of cancer cachexia, even though the exact underlying mechanisms remain to be elucidated. We investigated inflammation and partition of lipids in the liver of colorectal cancer weight stable (WSC) and colorectal cancer cachectic patients (CC) (Evans, 2008).


**Methods:** Nine WSC and nine CC participated in the study and seven patients with colelytiasis as a control groups (C). Liver biopsies were obtained during surgery. Liver samples were fixed in paraformaldeihyde and embedded into paraffin. The sections were stained with haematoxylin and eosin, PAS, and Mallory. Biopsies were evaluated in therms of histopathology, adopting the NASH activity score (Kleiner's score). After osmium impregnation, nine liver biopsies were studied under light and electron microscopy. Protein expression of inflammatory and chemotactic factors, and liver proteins were measured with Multiplex Magpix (®) system. Gene expression of liver lipid metabolism proteins was measured by qRT‐PCR. Student's *t*‐test or Mann–Whitney test with multiple comparisons was employed for parametric and non‐parametric data, respectively. The significance level was set at *P* < 0.05.


**Results:** Light histology and electron microscopy showed abundance of lipid droplets, indicating liver TAG accumulation in cachectic patients, as well as fibrosis, but NAFLD score yielded no differences among groups, despite increased portal activity in CC. Liver FABP mRNA was higher in WSC, compared with other groups (*P* = 0.0021). Protein expression of IL‐1α and IL‐8 were higher in CC (*P* < 0.05), compared with control and WSC.


**Conclusions:** Liver inflammation is associated with cachexia and contributes to hepatic steatosis. CC liver expressed higher inflammatory cytokine and increased lipids inclusion content compared to WSC and control groups.


**3-22**



**Transcriptome analysis reveals potential cancer cachexia biomarkers in head and neck squamous cell cancer patients with low muscularity**


Diogo de Moraes^1^, Sarah Santiloni Cury^1^, Paula Paccielli Freire^1^, Grasieli de Oliveira, Érica Nishida Hasimoto^2^, **Geysson Javier Fernandez García**
^3^ and Silvia Regina Rogatto^4^ and Robson Francisco Carvalho^1^



^1^
*Institute of Biosciences, São Paulo State University (UNESP), Botucatu, Brazil;*
^2^
*Department of Surgery and Orthopedics, School of Medicine, São Paulo State University (UNESP), Botucatu, Brazil;*
^3^
*Faculty of Medicine, University of Antioquia, UdeA, Medellín, Colombia;*
^4^
*Department of Clinical Genetics, University Hospital, Institute of Regional Health Research, University of Southern Denmark, Vejle, Denmark*



**Background:** Cancer cachexia is a multifactorial syndrome characterized by an ongoing loss of skeletal muscle mass that leads to increased morbidity and poor prognosis. The incidence of cachexia in patients with head and neck squamous cell carcinoma (HNSC) is unknown, but more than 50% of patients with advanced head and neck cancer have significant weight loss and possible cachexia. The molecular mechanism of cachexia in HNSC is incompletely understood, and there is no biomarker to predict which patients will develop the syndrome.


**Methods:** Here, we reanalysed computed tomography (CT) images—available on The Cancer Imaging Archive database—and tumour transcriptome data from The Cancer Genome Atlas (TCGA) aiming to identify new potentially secreted molecules by the tumours of patients with low muscularity. Sternocleidomastoid muscle area from CT of 66 HNSC patients (training set) was analysed to identify patients with high or low muscularity. Next, we studied the relationship of muscularity to overall survival and disease‐free survival. Moreover, to verify the biomarkers prognostic value in HNSC, we also used tumour gene expression data to predict survival using additional validation sets (four cohorts, 721 patients) available in SurvExpress database.


**Results:** Muscularity successfully discriminated HNSC patients into high‐risk and low‐risk groups, based on overall survival (*P* = 0.0120). Using tumour gene expression data from our training set, we identified 413 deregulated transcripts in patients with low muscularity. Genes encoding predicted secreted proteins such as *IL‐6*, *IL‐8*, *IL‐24*, *CCL24*, *FGA*, *FGB*, and *FGG* were found upregulated genes and are new potential cachexia biomarkers in HNSC secretome. Noteworthy, these tumour biomarkers were capable of distinguishing HNSC patients with poor prognosis.


**Conclusions:** Our integrative analysis of muscularity CT‐based data and transcriptome profiles identified cancer patients with low muscularity, from which the tumours expressed a set of cachexia‐related transcripts capable of predicting poor prognosis.


**3-23**



**Lipocalin 2 is a driver of sickness response during pancreatic cancer cachexia**



**Brennan Olson** and Xinxia Zhu and Peter Levasseur and Mason Noorgard and Kevin Burfeind and Katherine Michaelis and Daniel Marks


*Oregon Health & Science University, Portland, OR, USA*



**Background:** Cancer cachexia is a devastating condition that occurs in up to 80% of patients with pancreatic ductal adenocarcinoma (PDAC), where it significantly contributes to reduced survival, accelerated disease progression, and limits patients' ability to tolerate therapy. Effective therapies are lacking for cachexia, and its mechanisms remain elusive. Lipocalin 2 (LCN2) is an acute phase protein that mediates inflammation in several pathologic conditions and appetite during normal physiology but is unexplored in cancer cachexia. In this study, we assessed the role of LCN2 in driving sickness responses during the development of pancreatic cancer cachexia.


**Methods:** A pancreatic tumor cell line from a syngeneic C57BL/6 KRAS^G12D/+^ P53^R172/+^ Pdx‐Cre (KPC) mouse was orthotopically implanted into sex, age, and body weight‐matched wild‐type (WT) and LCN2 knockout (LCN2KO) mice. We monitored the effects of LCN2 deletion on PDAC cachexia through behavioural, molecular, and histologic analysis.


**Results:** LCN2 was robustly upregulated in the serum and cerebrospinal fluid of WT mice during PDAC cachexia, and brain endothelium amplify the expression of LCN2 when challenged with KPC conditioned medium *in vitro*. Central administration of LCN2 is sufficient to induce cachexia features of anorexia and weight loss. Finally, genetic deletion of LCN2 significantly ameliorated PDAC‐associated anorexia, fatigue, and muscle catabolism in both skeletal and cardiac muscles.


**Conclusions:** LCN2 is robustly induced in the periphery and brain during PDAC cachexia and is a critical driver of anorexia, muscle catabolism, and fatigue. Our findings implicate LCN2 as a pathologic mediator of cachexia symptoms and demonstrate its promise as a novel therapeutic target for this crippling condition.


**3-24**



**Colorectal cancer‐released exosomes are enriched in Hsp70 in cachectic patients**


Rodrigo S.C. De Sousa^1^, Joanna D.C.C. Lima^1^, Estefania Simoes^1^, Fang Chia Bin^2^, Fernanda Formiga^2^, Flavio Tokeshi^3^, Jose P. Otoch^3,4^, Paulo S.M. Alcantara^3^ and **Marialia Seelaender**
^1,4^



^1^
*Department of Cell Biology, University of Sao Paulo, Sao Paulo, Brazil;*
^2^
*Santa Casa da Misericórdia de São Paulo, Sao Paulo, Brazil;*
^3^
*Hospital Universitário, Universidade de São Paulo, Sao Paulo, Brazil;*
^4^
*Clinical Surgery, Faculty of Medicine, Sao Paulo, Brazil*



**Background:** Cachexia is an inflammatory syndrome characterized by muscle wasting that leads to an increased cancer mortality due to weight loss, low quality of life, anorexia, and systemic inflammation. The mechanism about cancer‐induced weight loss remains unclear. Emerging evidence support that tumour‐released heat shock proteins, such as Hsp70 and Hsp90, are responsible for the tumour capacity to cause muscle loss. The aim of this study is to investigate levels of Hsp70 and Hsp90 released by tumour‐derived exosomes from colorectal cachectic patients.


**Methods:** Colorectal cancer patients were divided into weight stable cancer (WSC *n* = 10) and cachectic cancer (CC *n* = 10) groups, after signature of the informed consent form. Samples were collected during surgery. Exosomes were isolated from tumour tissue explants culture and characterized based on size and morphology by transmission electron microscope (TEM). Western blot employing typical exosome markers (CD63) was performed. Hsp70 and Hsp90 levels in tumour‐derived exosomes and plasma were analysed by ELISA following the manufacturer's instruction.


**Results:** Particles were confirmed to be exosomes by TEM; Exosomes presented typically cup‐shaped extracellular vesicles morphology with 30–100 nm diameter. In addition, we found expression of CD63+ exosomes detectable in a dose‐dependent assay (50, 12.5, and 3.1 μg). We detected high level of Hsp70 in tumour‐derived exosomes from cachectic patients (*P* = 0.022), although we did not find difference in Hsp90 expression in the exosomes between the groups (*P* = 0.955). Finally, we observed a tendency of Hsp90 content to be increased in the circulation of cachectic cancer patients (*P* = 0.075).


**Conclusions:** In summary, the higher levels of tumour‐released heat shock proteins in cachectic patients suggest a new role of Hsp70 and Hsp90 as cachexins contributing to muscle wasting, as previously found for animal models. These findings may be related to the increased tumour secretion of inflammatory factors and the unbalance redox response mediated by hypoxic conditions in the tumour microenvironment of cachectic patients described by our group in a previous study.


**3-25**



**Biologically distinct body composition features in colorectal cancer**



**Victoria Armstrong**
^1^, Cynthia Stretch^1^, Liam Fitzgerald^2^, Jennifer Koziak^1^, Karen Kopciuk^1,3^ and Oliver F. Bathe^1,4^



^1^
*Department of Oncology, University of Calgary, Calgary, Canada;*
^2^
*Faculty of Kinesiology, University of Alberta, Edmonton, Canada;*
^3^
*Departments of Community Health Sciences, Mathematics and Statistics, University of Calgary, Calgary, Canada;*
^4^
*Department of Surgery, University of Calgary, Calgary, Canada*



**Background:** Cancer cachexia leads to marked alterations in body composition which can be captured using computed tomography (CT) imaging. Low muscle mass (sarcopenia), low muscle radiodensity (myosteatosis) and, more recently, high‐fat radiodensity have all been described in patients with cancer cachexia. However, it is not clear whether these represent different phenotypes driven by different biological phenomena.


**Methods:** In 340 patients with colorectal cancer (CRC) (stages 1–4), body composition features were evaluated on CT scans at L3 using Slice‐O‐Matic image analysis. Associations with overall survival (OS) were explored, using Cut‐off Finder. Blood sera from these patients were analysed for 20 proteins implicated in cancer‐associated alterations in metabolism and inflammation.


**Results:** We determined the skeletal muscle index, muscle radiodensity, and subcutaneous fat density associated with a decreased OS in this cohort. Specifically, we defined sarcopenia, myosteatosis, and high‐fat density by dichotomizing patients with optimal cut‐off values associated with significantly decreased OS using log‐rank testing (*P* = 0.010, *P* = 0.048, and *P* = 0.001, respectively). Using population‐specific cut‐offs, 44% had sarcopenia; 16% had myosteatosis; and 33% had high‐fat density. These features appeared mutually exclusive; 57% of patients had only one of these features. OS was even more truncated when more than one of these body composition features coexisted (*P* < 0.001). Patterns of circulating mediators were significantly different in individuals with each body composition feature. Briefly, sarcopenia was associated with low levels of IGF‐1 (*P* = 0.001), myosteatosis was associated with high levels of leptin (*P* = 0.001), and high‐fat density was associated with high levels of adiponectin (*P* = 0.005).


**Conclusions:** Three body composition features associated with reduced OS were observed in CRC patients. These appeared to be mutually exclusive, biologically distinct, and have synergistic effects on survival. Currently, we are evaluating the serum lipidome and metabolome as well as broad‐spectrum proteomics of this cohort to identify additional correlates that will inform underlying biological associations.


**3-26**



**Risk factors for surgery‐related muscle loss after liver resection for colorectal liver metastasis**



**Laura van Wijk**
^1^, Stijn van Duinhoven^1^, Alain R. Viddeleer^2^, Mike Liem^3^, Donald E. Bouman^4^ and Joost M. Klaase^1,3^



^1^
*Department of Hepatobiliary Surgery and Liver Transplantation, University Medical Center Groningen, Groningen, The Netherlands;*
^2^
*Department of Radiology, University Medical Center Groningen, Groningen, The Netherlands;*
^3^
*Department of Surgery, Medisch Spectrum Twente, Enschede, The Netherlands;*
^4^
*Department of Radiology, Medisch Spectrum Twente, Enschede, The Netherlands*



**Background:** Preoperative sarcopenia and surgery‐related muscle loss (SML) negatively affect postoperative outcome. Although the impact of preoperative sarcopenia has been well described, limited literature is available about SML and its risk factors after liver resection. By identifying these risk factors, perioperative intervention might prevent or reduce SML and subsequently improve postoperative outcome. This study investigated risk factors for (clinically relevant) SML and outcome after liver resection for colorectal liver metastasis.


**Methods:** We retrospectively analysed data of patients diagnosed with CRLM who underwent liver resection from 2006 to 2016. Total psoas area (TPA) muscle index was measured using computed tomography images at L3 level, obtained within 6 weeks before and 6 weeks after surgery. Change in TPA after surgery was calculated. Muscle loss ≥5% was defined as clinically relevant SML.


**Results:** A total of 121 patients were analysed. Fifty‐five (45%) patients had SML of whom 32 (58%) had clinically relevant SML. Multivariate analysis demonstrated that diabetes (*P* = 0.027) and a preoperative high TPA muscle mass index (*P* = 0.039) were associated with SML. However, a preoperative low muscle mass index (*P* = 0.003), diabetes (*P* = 0.019), pulmonary disease (*P* = 0.002), and male gender (*P* = 0.026) were associated with clinically relevant SML. Clinically relevant SML was associated with a decreased overall survival (*P* = 0.029).


**Conclusions:** Almost half of patients had SML within 6 weeks after liver resection for CRLM. Among them, nearly 60% had clinically relevant SML. A preoperative low muscle mass index, diabetes, pulmonary disease, and male gender were found to be independently associated with clinically relevant SML. Clinically relevant SML had a negative effect on overall survival.


**3-28**



**Role of mTOR in skeletal muscle during cancer cachexia**



**Alessia Geremia**
^1^, Martina Baraldo^1^, Valeria Balmaceda Valdez^1^, Roberta Sartori^1^, Clara Turk^2^, Simona Boncompagni^3^, Marcus Kruger^2^ and Bert Blaauw^1^



^1^
*Venetian Institute of Molecular Medicine, Padova, Italy;*
^2^
*University of Cologne, Cologne, Germany;*
^3^
*University of Chieti, Chieti, Italy*


Cancer cachexia is a multiorgan syndrome which is characterized by a major loss in body weight, particularly in muscle and adipose tissue. It has been shown that if this cancer‐related muscle wasting is reserved or prevented, lifespan is significantly improved. A critical mediator of adult muscle mass and function in skeletal muscle is the kinase mTOR; however, its role during cancer cachexia is unknown yet.

To assess the function of mTOR during cancer cachexia, we used two mouse models in which we have deleted mTOR or Raptor only in adult skeletal muscle; in the first one, both mTOR complexes are absent, instead, in the Raptor model, only mTORC1 result deleted. We performed two models of cancer cachexia: Lewis Lung Carcinoma (LLC) that leads to chronic cachexia and C26 Colon Carcinoma that leads to acute atrophy.


**3-29**



**Investigation of the direct role of tumour secretions in the development of cachexia in head and neck cancer patients**


Nicolas Saroul^1^, Stephane Walrand^2^, Yves Boirie^2^, Olav Rooyackers^3^ and **Nicolas Tardif**
^3^



^1^
*ENT Department, CHU de Clermont‐Ferrand, Clermont‐Ferrand, France;*
^2^
*INRA, UMR1019, Université Clermont Auvergne, UNH, Unité de Nutrition Humaine, CRNH Auvergne, Clermont‐Ferrand, France;*
^3^
*Anesthesiology and Intensive Care, Department of Clinical Science Intervention and Technology (CLINTEC), Karolinska Institutet, Huddinge, Sweden*



**Background:** Head and neck cancer (HNC) patients present a high prevalence of cachexia and malnutrition at diagnosis. Contributions of malnutrition versus the direct role of tumour secretions in the development of cachexia are still unclear in HNC patients. We have investigated the role of tumour‐secreted factors in loss of skeletal muscle in HNC patients.


**Methods:** At 7 days of differentiation, human primary myotubes were incubated for 48 h with serum from HNC patients (*n* = 25) and controls (*n* = 14) or with conditioned media (CM) from two HPV human squamous cell carcinoma (HPV group) or a control CM (CMC group). At inclusion, HNC patients muscle mass was assessed by CT scan. Except for mitochondria respiration assays, due to limited volume, patients' serum were mixed in three groups: mix of controls (C, *n* = 14), mix of patient with low degree of sarcopenia (LS, *n* = 10), and mix of patient with severe sarcopenia (SS, *n* = 10).


**Results:** Basal mitochondrial respiration was 28% lower in myotubes incubated with serum from HNC patients (*P* = 0.008 vs. C). Mitochondrial biogenesis markers were unchanged in HPV group and patients' groups. MnSOD gene expression was significantly higher in SS and HPV groups compared to their respective controls. MyHC protein level was 50% lower in HPV‐treated myotubes (*P* = 0.0005). MyHC level was unaltered in SS‐incubated myotubes and 43% higher in LS‐incubated myotubes (*P* = 0.003; C vs. LS). A 26S proteasome activity was unchanged in all tested conditions. A 31% higher phosphorylation of p70S6K (Thr‐389) was measured in LS‐incubated myotubes (*P* = 0.008; C vs. LS). We are currently assessing the protein synthesis rate (SUNSET method).


**Conclusions:** In our model, HNC patients' serum are able to decrease basal mitochondrial respiration without inducing atrophy. In this model, we cannot conclude yet if mitochondrial impairments are an early event in atrophy development or if atrophy and mitochondrial impairment are independently regulated.


**4-07**



**A machine learning approach for cachexia diagnosis**



**Natasha Fioretto Aguero**
^1^, Gabriela Salim de Castro^2^, Danilo Lessa Bernardineli^1^, Marília Seeländer^2^ and Alexandre Alarcon do Passo Suaide^1^



^1^
*Instituto de Física da Universidade de São Paulo, Sao Paulo, Brazil;*
^2^
*Instituto de Ciências Biomédicas USP, Sao Paulo, Brazil*


The diagnosis of cancer‐associated cachexia before the manifestation of its most common symptoms, such as muscle wasting, is a major challenge nowadays. If feasible, this could greatly influence identification and management of the syndrome and the patient's prognosis and quality of life. Recently, artificial intelligence techniques, such as machine learning, have been used in health sciences to allow early and more precise diagnosis of several diseases. In this work, we explore a machine learning algorithm to evaluate clinical and biochemical data from patients with gastrointestinal cancer with clear symptoms of cachexia (CC group), weight stable cancer patients (WSC group), and healthy volunteers, that is, without being diagnosed with cancer (Control group). We employed the K‐means algorithm to divide the data into two groups in an unsupervised approach, which means that the patients' previous group allocation was not known by the program. We created four different training models, ranging from models with a huge amount of features to simpler ones with only four biochemical parameters. In each model, the program was able to identify two distinct clusters: one allocated the majority of healthy patients and the other had the majority of patients with cancer cachexia. Then, patients with weight stable cancer were subjected to these classification tests; part of them were labelled by the computer as having biochemical alterations compatible with the cachexia group. Could this be indicative of very early stages of cachexia? In this work, we discuss the employed machine learning method and compare the proposed models regarding their accuracy, precision, and recall. Furthermore, the advantages of using a very small set of biochemical data will be approached as a way to identify patients with cancer in precachexia stages.


**4-09**



**A multiple‐plasma free amino acid signature predicts cachexia symptoms during first‐line chemotherapy for advanced pancreatic cancer**



**Shuichi Mitsunaga**
^1,2^, Michihiro Takada^3^, Sachiko Nishikawa^3^, Akira Imaizumi^3^, Masafumi Ikeda^1^ and Atsushi Ochiai^2^



^1^
*Department of Hepatobiliary & Pancreatic Oncology, National Cancer Center Hospital East, Kashiwa, Japan;*
^2^
*Research Center for Innovative Oncology, National Cancer Center, Kashiwa, Japan;*
^3^
*Research Institute for Bioscience Products & Fine Chemicals, Ajinomoto Co., Inc., Kawasaki, Japan*



**Background:** The combining number of cancer cachexia symptoms (CACO) is an index of the severity of cancer cachexia (CAC). Circulating plasma levels of free amino acids (PFAAs) are known to be influenced by CAC and also by the presence of ductal adenocarcinoma of pancreas (PDAC) and could be a predictive biomarker of CACO. This study was aimed at building and validating a signature comprised of multiple PFAAs for discriminating severe CACO from nonsevere CACO during first‐line chemotherapy for advanced PDAC.


**Methods:** A single‐centre retrospective study on the diagnostic performance of multiple PFAAs was conducted. Patients (pts) with treatment‐naïve PDAC whose frozen plasma samples were available were enrolled. The target population was divided into the training or validation cohort at an allocation ratio of 5:3. Symptom of CAC was defined as body weight loss ≥2% in the previous 6 months, Karnofsky performance status (KPS) ≤80, appetite interference score ≥4 according to the Japanese version of the MD Anderson symptom inventory, or serum C‐reactive protein level ≥0.5 mg/dL, and was evaluated at 1 month after the start of first‐line chemotherapy. Patients with three or more symptoms of CAC were classified as having severe CACO. A total of 19 PFAAs were measured using liquid chromatography–mass spectrometry. An index consisting of the PFAAs at the baseline was evaluated for its ability to predict severe CACO 1 month later.


**Results:** Data of a total of 160 pts with treatment‐naïve advanced PDAC (100 in the training set and 60 in the validation set) were analysed in this study. Severe CACO at 1 month after the baseline was observed in 17% of the training cohort and 30% of the validation cohort. In the training set, 40 349 signatures consisting of two to six PFAAs were developed, and 322 multiple‐PFAA combinations were identified as the diagnostic signature. For the best‐performing PFAA signature, the area under the curve in the validation cohort was 0.807 (95% confidence interval, 0.685–0.930).


**Conclusions:** A multiple PFAA index is a promising biomarker for the prediction of CAC symptoms during first‐line chemotherapy for advanced pancreatic cancer.


**4-10**



**Low BMI obesity and increased myopenia in colorectal cancer patients with education and employment deprivation in the England: two sides of the same coin?**



**Edward Tobias Pring**
^1,2,3,4^, Laura E. Gould^1,2^, Georgios Malietzis^1,3^, Thanos Athanasiou^1,3^ and John T. Jenkins^1,2,3^



^1^
*George Davies Research Fellowship, Leicester, UK;*
^2^
*BiCyCLE Research Group, St. Mark's Hospital, Harrow, UK;*
^3^
*Department of Surgery, St. Mark's Hospital, Harrow, UK;*
^4^
*Imperial College London, London, UK*



**Introduction:** Adults living in the most deprived areas of England are 46% more likely to be obese than those in the least deprived areas. Little is known about the effect of deprivation on cancer cachexia. Patients with increased cachexia have a worse outcome from cancer, specifically in colorectal cancer (CRC) myopenia is associated with worse outcomes following surgery. We investigated the effect of employment and education deprivation on the body composition of patients with CRC.


**Methods:** A prospectively maintained database of primary CRC patients undergoing surgery at a UK specialist bowel hospital. Postal codes were used to determine the level of deprivation of each CRC case using England's index of multiple deprivation database. Preoperative computer tomographic body composition (CTBC) analysis was performed using SliceOmatic v5.0 and ABACS L3. Cut‐off values for body composition variables were used from previous work by Prado *et al* (2008) and Doyle *et al* (2013).


**Results:** Myopenia was significantly associated with employment *P* = 0.017, OR 1.23, (95% CI 1.04–1.46) and education *P* = 0.034, OR 1.25, (95% CI 1.02–1.54) deprivation.

BMI Obesity (BMI > 30) was significantly associated with those with the least deprived in terms of employment *P* = 0.004, OR 1.18, (95% CI 1.05–1.33) and education *P* = 0.001, OR 1.17, (95% CI 1.05–1.29) deprivation. There was no significant relationship between visceral obesity and employment *P* = 0.117, OR 1.07, (95% CI 0.98–1.17) or education *P* = 0.19, OR1.05, (95% CI 0.98–1.13) deprivation. A clinically significant neutrophil‐to‐lymphocyte ratio (NLR > 3) was associated with employment (*P* = 0.009) and education (*P* = 0.038) deprivation. Whilst a clinically significant raised platelet‐to‐lymphocyte ratio (PLR > 130) was associated with employment deprivation alone (*P* = 0.008).


**Conclusions:** Myopenia and systemic inflammation are associated with education and employment deprivation in CRC. BMI obesity is associated with CRC patients with the least education and employment deprivation. However, this is not reflected in visceral obesity where no significant difference exists between either group. BMI obesity fails to truly reflect the cachectic picture which appears markedly worse in the most deprived cancer patients.


**4-11**



**Myosteatosis is an independent predictor of distant metastatic disease in colorectal cancer: a potential biomarker to guide enhanced surveillance and earlier treatment**



**Edward Tobias Pring**
^1,2,3,4^, Laura E. Gould^1,2^, Georgios Malietzis^1,3^, Thano Atanasiou^1,3^ and John T. Jenkins^1,2,3^



^1^
*George Davies Research Fellowship, Leicester, UK;*
^2^
*BiCyCLE Research Group, St. Mark's Hospital, Harrow, UK;*
^3^
*Department of Surgery, St. Mark's Hospital, Harrow, UK;*
^4^
*Imperial College London, London, UK*



**Background:** There are over 42,000 cases of colorectal cancer per year in the UK, with over 16,000 deaths from the disease per year. A total of 30% to 40% of the patients develop recurrent disease and most of these die from their disease. Early detection of recurrent disease at a presymptomatic stage may allow expedient treatment and increase survival. We aimed to determine whether body composition could be a used to predict recurrent disease.


**Method:** Analysis was performed on a prospectively maintained database of primary colorectal cancer patients undergoing surgery at a single UK specialist bowel hospital. Preoperative computer tomographic body composition analysis was performed using SliceOmatic v5.0 and ABACS L3. Cut‐off values for body composition variables were used from previous work by Prado *et al* (2008) and Doyle *et al* (2013). Univariate and multivariate logistic regression analyses were performed on these data.


**Results:** A total of 1401 patients were included, median age 69 years [IQR 60–77] and 57% male. On univariate analysis, myosteatosis (*P* = 0.013) and visceral obesity (*P* = 0.006) were found to be significantly associated with distant disease whilst myopenia (*P* = 0.668), sarcopenic obesity (*P* = 0.675), and BMI obesity (*P* = 0.901) were not. A total of 633 patients were included in the multivariate model; at the time of surgery, 10% (61) patients had poorly differentiated tumours; 69% (441) were T3/4; 41% (261) had positive nodes; and 23% (145) patients had or developed distant metastatic disease. Preoperatively, 59% (337) were viscerally obese and 74% (470) myosteatotic. Myosteatosis (*P* = 0.001), positive lymph nodes (*P* = 0.0001), and T3/4 tumours (*P* = 0.004) were independently associated on multivariate regression analysis with distant disease.


**Conclusions:** Myosteatosis is an independent predictor of the presence of distant metastases and may even presage metastatic disease before it can be identified on imaging. This may allow clinicians to target surveillance and earlier intervention towards those patients who are at risk of recurrent disease, potentially preventing or reducing the rate of disease progression.


**4-12**



**Anaemia in children with end‐stage chronic liver disease**


Jenny Ashworth^1^, Kar Yan‐Yip^1^ and **Eirini Kyrana**
^1,2^



^1^
*Medical School, University of Leeds, Leeds, UK;*
^2^
*Children's Liver Unit, Leeds Children's Hospital, Leeds, UK*



**Background:** Anaemia of chronic liver disease and therefore end‐stage chronic liver disease (ESCLD) has been described in adults and has been associated with more severe liver disease. It has not been specifically studied in children.


**Methods:** Retrospective review of all children who had a liver transplant at our unit between 2013 and 2017 (inclusive). The aim was to identify the incidence of anaemia and its relationship with the disease severity in children receiving a liver transplant for ESCLD. A level of haemoglobin <10.5 g/L was considered as low.


**Results:** Our database identified 80 children (40 F: 40 M). Mean age was 5.2 years (range 0.3–15.88 years, median 2.9 years). Of those, 58 had ESCLD (mean age 5.48 years, range 0.31–15.88 years, and median 1.13 years). Forty‐one out of 80 of the patients overall, where anaemic prior to liver transplant and in particular 27/58 of the ones with ESCLD, were anaemic. Of these, 19/27 had a normocytic anaemia, six a macrocytic, and two a microcytic. The anaemic children with ESCLD were significantly younger, had a higher bilirubin and were more coagulopathic, and had a higher PELD (paediatric end‐stage liver disease) score than the nonanaemic ones. Anaemia did not correlate with white cell and platelet count (i.e. the presence of hypersplenism due to portal hypertension). The anaemic children had a significantly lower mean weight z‐score than the nonanaemic ones (−1.7 versus −0.69, *P* < 0.05). A total of 51.8% of the anaemic children had a weight z‐score ≤1.96, in comparison to 16.1% of the ones without anaemia. Anaemia did not correlate with length of stay in hospital after liver transplant.


**Conclusions:** The presence of anaemia in children with ESCLD is associated with the severity of the disease and the growth impairment.


**4-13**



**Sarcopenia, myopenia, phase angle in cancer: a cut‐off to optimize nutrition efficacy?**


Pedro Miguel Neves^1^, Filomena Pina^2^, Isabel Diegues^2^, Céu Raimundo^2^, Inês Almada Correia^1^, Mariana Tomaz^1^, Pedro Marques Vidal^3^ and **Paula Ravasco**
^1,4,5^



^1^
*Centro de Investigação Interdisciplinar em Saúde da Universidade Católica Portuguesa, Lisbon, Portugal;*
^2^
*Serviço de Radioterapia do Hospital Universitário de Santa Maria, CHULN, Lisbon, Portugal;*
^3^
*CHUV and Faculty of Biology and Medicine, Institute of Social and Preventive Medicine (IUMSP), Lausanne, Switzerland;*
^4^
*Hospital Universitário de Santa Maria, CHULN, Lisbon, Portugal;*
^5^
*University of Lisbon, Portugal*



**Background:** Poor nutritional status in cancer is mainly manifested by severe muscle mass depletion, that may happen at any stage of cancer treatments (curative, adjuvant, and palliative), independently of body weight. Evidence shows that sarcopenia/myopenia detrimentally impacts clinical outcomes; studies suggest a role of phase angle undernutrition diagnosis.


**Methods:** Ongoing study to include 400 cancer patients: gastrointestinal, lung, head–neck referred for radiotherapy. Parameters: PG‐SGA, body composition + phase angle (PA) (BODYSTAT®), dietary intake, quality of life (QLQ‐C30), symptoms/toxicity, physical function (Karnofsky, ECOG).


**Results:** In 63 patients(18F:45M), PA was below age/sex reference in 37/48 men and 13/18 women; PA fell 1 integer below lower limit in 27pts and fell 2 integers in 16pts. Coincidentally, 34/63pts with lowest PA had worse PG‐SGA scores (>9points) and tended to have worse ECOG (*P* = 0.06). In men, PA and physical function were strongly correlated (*P* < 0.001). Lower FFMI was seen in patients with PA with two integers below lower limit and with worse ECOG (*P* < 0.001). Lower FFMI + PA correlated with nutrition impact symptoms (*P* < 0.001). Notably, protein intake was <1.5–1.4 g/kg/d in 3pts; ≤1.3–1.1 g/kg/d in 16pts; ≤1.0–0.6 in 24pts, ≤0.5 in 10pts; in 10pts intake ≈recent guidelines (≥1.5 g/kg/d). Most severe deficits (≤1.0–0.4 g/kg/d) were present in patients with lower PA (0.001) and FFMI (0.002).


**Conclusions:** In this cohort, significant impairments in body composition, PA, function, and symptom severity were present and interrelated already at diagnosis. The proposal of a cut‐off for sarcopenia/myopenia/PA with an easy to use, cost‐effective, globally available method could abbreviate a tailored intervention to prevent/reverse/treat further nutritional wasting. Conscious of the limitations intrinsic to sample size and BIA parameters, PA with 1–2 integers below lower limit combined with low FFMI, seemed effective in discriminating baseline wasting and in predicting function impairment and severe protein deficits. In the absence of CT scans to assess body composition, BIA may prove determinant and outperforms subjective methods. Given the role of body composition in oncological outcomes, strategies to optimize intervention are important for a successful cancer therapy.


**5-05**



**Adverse muscle composition within obesity is associated with low functional performance and increased comorbidity: results from the large UK Biobank imaging study**



**Jennifer Linge**
^1,2^ and Olof Dahlqvist Leinhard^1,2,3^



^1^
*AMRA Medical, Linköping, Sweden;*
^2^
*Department of Medical and Health Sciences, Linköping University, Linköping, Sweden;*
^3^
*Center for Medical Image Science and Visualization (CMIV), Linköping University, Linköping, Sweden*



**Background:** Sarcopenia within obesity is not well described. Recent results based on the UK Biobank showed only 0.1% of participants with obesity had sarcopenia (EWGSOP2 criteria) while contradictory, they showed the highest prevalence of low functional performance^1^. The main cause for under diagnosis of sarcopenia within obesity is the BMI‐dependency of current sarcopenia definitions, where the correlation between body size and muscle mass has not been properly adjusted through division with, e.g., height^2^, weight, or BMI^1^.


**Methods:** A total of 9612 participants were included (*N* = 4589 with DXA). Fat‐free muscle volume (FFMV) and muscle fat infiltration (MFI) were quantified using a 6 min MRI protocol and automated image analysis (AMRA® Researcher). For each participant, a sex‐and‐BMI matched virtual control group (VCG) was created. As a measure of deviating FFMV, the individual FFMV/height^2^ z‐score was extracted from each VCG‐distribution (FFMV_VCG_)^1^. Participants with obesity (BMI ≥ 30 kg/m^2^) and adverse muscle composition were stratified using sex‐specific thresholds for MFI (above 75th percentile, whole cohort) and FFMV_VCG_ (below 25th percentile, whole cohort). The functional performance (hand grip strength, walking pace, stair climbing, and falls) and comorbidity (coronary heart disease and type 2 diabetes) in participants with obesity and adverse muscle composition were compared to those without adverse muscle composition. As reference, characteristics of the sarcopenia population (stratified through EWGSOP2 criteria using the DXA‐subset) were also included.


**Results:** A total of 311 out of 1808 participants with obesity had adverse muscle composition (prevalence within obesity 17.2%). The prevalence of low functional performance was significantly higher in participants with adverse muscle composition for all variables (all *P* < 0.05, age‐adjusted) except falls (non‐significant) (Table 1). The prevalence of comorbidities was significantly higher in participants with adverse muscle composition (all *P* < 0.01, age‐adjusted) (Table 1).

**Table 1 jcsm12551-subcmp-0023-tbl-0001:** Characteristics of participants with obesity with (w/) and without (w/o) adverse muscle composition showing mean (SD) and prevalence including level of significance

	Groups for statistical comparison		Reference data	
	Obesity w/ adverse muscle composition	Obesity w/o adverse muscle composition	Sarcopenia (EWGSOP2) in DXA subset	Whole cohort
N participants	311	1497	102	9612
% females	50.5%^a^	51.9%	77.5%	52.5%
Age (years)	66.1 (6.7)***	61.2 (7.3)	65.6 (6.9)	62.6 (7.5)
Weight (kg)	92.8 (12.4)^a^	94.1 (13.4)	61.3 (8.9)	75.5 (14.8)
BMI (kg/m^2^)	33.5 (3.1)^a^	33.5 (3.4)	23.1 (2.5)	26.6 (4.4)
Sarcopenia (EWGSOP2) in DXA subset	0.7%^a^	0.0%	100%	2.2%
Low hand grip strength	11.3%*	5.7%	100%	6.4%
Slow walking pace	23.8%***	9.2%	9.8%	4.4%
No stair climbing	16.4%**	8.7%	8.8%	7.9%
More than 1 fall last year	8.7%^a^	6.7%	8.8%	4.8%
Coronary heart disease	15.4%**	6.4%	7.8%	4.6%
Type 2 diabetes	19.3%***	9.0%	1.0%	4.5%

a non‐significant,

*
*P* < 0.05,

**
*P* < 0.01,

***
*P* < 0.001, age‐adjusted.


**Conclusions:** Adverse muscle composition within obesity, as identified using MRI, is commonly observed and associated with high prevalence of low functional performance and comorbidities.

## References


1. 
Linge
J
, 
Heymsfield
SB
, 
Dahlqvist
Leinhard O
. On the definition of sarcopenia in the presence of aging and obesity—initial results from UK Biobank. J Gerontol A Biol Sci Med Sci
2019 (in press).10.1093/gerona/glz229PMC730218131642894



**5–24**



**Low‐magnitude, high‐frequency vibration treatment attenuates age‐related neuromuscular junction degeneration**


Bao Zhengyuan and Cui Can and Qin Ling and Cheung Wing‐Hoi and **Chow Simon Kwoon‐Ho**



*Musculoskeletal Research Laboratory, Department of Orthopaedics & Traumatology, Faculty of Medicine, The Chinese University of Hong Kong, Shatin, Hong Kong*



**Background:** Sarcopenia is a phenomenon characterized by age‐related decline in muscle mass and strength. There are multiple aetiological factors leading to sarcopenia, and neuromuscular junction (NMJ) degeneration is among one of the causes. According to our previous studies, whole‐body low‐magnitude high‐frequency vibration (LMHFV) treatment could improve skeletal muscle function in sarcopenia, but the mechanisms are unclear, so this study aims to investigate the effects of LMHFV on NMJ degeneration in sarcopenia.


**Methods:** Senescence‐accelerated mouse prone 8 (SAMP8) were previously characterized to exhibit sarcopenic phenotype. A total of 54 male mice aged 6 months were randomized into control (Ctrl) and vibration treatment (VT) groups. The mice in the VT group were treated with LMHFV (35 Hz, 0.3 g, where g = gravitational acceleration) 20 min/day and 5 days/week. NMJ *ex vivo* function and structure were evaluated at months 0, 2, 3, 4, and 6 post‐treatments with six mice in each time point. Student's *t*‐test was used for treatment effect considered at *P* < 0.05.


**Results:** In NMJ *ex vivo* function tests, specific tetanic force in VT group at Month 3 post‐treatment increased by 15% compared with Ctrl group. Morphologically, immunofluorescence of the whole‐mount aged muscle specimens showed significant fragmentation of the characteristic pretzel structure. Quantitative results showed that discontinuity index of NMJ postsynaptic acetylcholine receptors in Ctrl group was higher than that in VT group at Month 4 post‐treatment (10 in Ctrl versus 7.8 in VT group) with statistical difference.


**Conclusions:** LMHFV was previously shown to enhance muscle function in sarcopenic mice. Current results suggest that LMHFV treatment could achieve the enhancement through improving NMJ function and attenuate morphological degeneration of the NMJ in sarcopenic animal model during ageing suggesting that vibration is a promising treatment to tackle muscle denervation is aged muscles.


**5-25**



**Mid‐arm muscle circumference is an anthropometric all‐cause mortality's predictor for noninstitutionalized elderly: Cohort Elderly Project/Goiania**


Cristina Camargo Pereira^1^, Valéria Pagotto^2^, Annelisa Silva and Alves de Carvalho Santos^3^ and Erika Aparecida Silveira^1^



^1^
*Health Sciences Graduate Program, School of Medicine, Universidade Federal de Goiás, Goiânia, Brazil;*
^2^
*Nursing Graduate Program, Universidade Federal de Goiás, Goiânia, Brazil;*
^3^
*Faculdade Unida de Campinas, Goiânia, Brazil*



**Background**: Mid‐arm muscle circumference (MAMC) is an anthropometric indicator of muscle mass, which has been used in nutritional status assessment and risk prediction. However, its association with mortality in the elderly population remains unknown. We investigated the impact of low MAMC on mortality risk in noninstitutionalized elderly individuals.


**Methods**: Cohort study Elderly Project/Goiania, which evaluated 418 noninstitutionalized elderly (≥60 years old) living in Goiania's metropolitan area, capital of Goias state, Brazil. MAMC was calculated using the standard formula: MAMC = mid‐arm circumference – (0.314 × triceps skinfold thickness). We categorized both male and female participants into tertiles based on their MAMC level. Low MAMC was defined as the lowest sex‐specific tertile. Sociodemographic variables included age and gender. Data mortality was collected from the Brazilian Mortality Information System of the Health Ministry. We used Cox proportional hazards regression analyses to estimate the hazard ratios (HRs) for all‐cause mortality. *P* values less than 0.05 were considered statistically significant.


**Results**: During a mean of 8.5 years of follow‐up period, we observed 144 deaths from all‐causes, 39.7% of the total cohort sample. We included 416 elderly being 66.0% female participants and an average age of 70.7 ± 7.1 years. The mean MAMC was 25.8 ± 2.7 cm in men and 23.6 ± 3.0 cm in women (*P* < 0.01). Low MAMC was observed in 14.1% (95% CI: 8.29–19.87%) of men and 43.3% (95% CI: 37.38–49.16) of women (*P* < 0.01). Cox proportional hazards regression analyses showed that low MAMC was associated with all‐cause mortality (HR = 1.70, 95% CI: 1.22–2.36) (*P* < 0.01) (Figure 1), with highest risk mortality among women (HR = 2.02, 95% CI: 1.32–3.09) (*P* < 0.01) than men (HR = 1.99, 95% CI: 1.02–3.86) (*P* < 0.04).

**Figure 1 jcsm12551-subcmp-0025-fig-0001:**
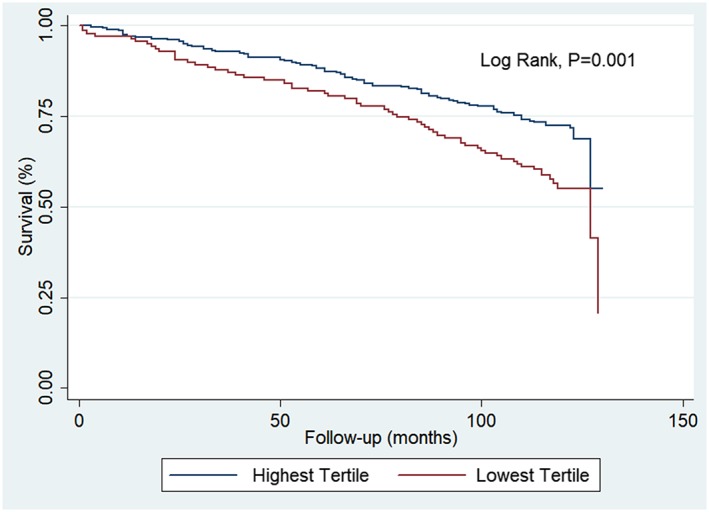
Kaplan–Meier survival curves stratified by mid‐arm muscle circumference (MAMC) status (highest tertile vs. lowest tertile).


**Conclusions**: Our study showed that MAMC is inversely associated with all‐cause mortality in Brazilian males and females noninstitutionalized elderly.


**5-26**



**The sex difference of relationship between sarcopenia and chronic kidney disease in older Korean adults**



**Soo Jeong Choi**
^1^, Min Sung Lee^1^, Su‐Hyun Kim^2^, Seok Hui Kang^3^, Ran‐Hui Cha^4^, Miyeun Han^5^ and Jun Chul Kim^6^



^1^
*Department of Internal Medicine, Soonchunhyang University Bucheon Hospital, Bucheon, South Korea;*
^2^
*Department of Internal Medicine, Chung‐Ang University Hospital, Seoul, South Korea;*
^3^
*Department of Internal Medicine, Youngnam University Hospital, Daegu, South Korea;*
^4^
*Department of Internal Medicine, National Medical Center, Seoul, South Korea;*
^5^
*Department of Internal Medicine, Pusan National University Hospital, Pusan, South Korea;*
^6^
*Department of Internal Medicine, CHA University Gumi Medical Center, Gumi, South Korea*



**Background:** Sarcopenia can affect the prognosis of patients with chronic kidney disease (CKD). CKD and sarcopenia increase with ageing. Males with CKD appear to be more prone to sarcopenia than females with CKD. However, few reports are available about the relationship between sex differences and sarcopenia and CKD stage in older people. The aim of this study was to examine the association between CKD and sarcopenia and sex differences in older adults.


**Methods:** This cross‐sectional study is based on the Korean Fraility and Ageing Cohort study, which involved a population of 2403 people aged 70 years of age or over. Anthropometric, physical performance, and baseline laboratory data were collected. CKD was defined by an estimated glomerular filtration rate (eGFR) < 60 mL/min/1.73m^2^ or proteinuria.


**Results:** The mean age of the participants was 76.0 ± 3.9 years old and 52.8% of them were female. The number of male and female CKD patients were 215 and 137, respectively (18.9 vs. 10.8%, *P* < 0.001). The eGFR was correlated with gait speed (*r* = 0.137, *P* < 0.001), sit to stand (STS) time (*r* = −0.074, *P* = 0.001), timed up and go (TUG) test (*r* = −0.156, *P* < 0.001). Appendicular skeletal muscle mass (ASM) index values were correlated with eGFR in male but not female participants. The share of participants with slow gait and STS and TUG increased according to CKD stage. Participants with low handgrip power were not different between men and women. The sex difference of relationship between physical performance and eGFR was the same after adjusting diabetes, weight ,and laboratory data.


**Conclusions:** Declines in ASM Index and physical performance except handgrip power increase according to the CKD stage in older people. CKD was associated with an increased prevalence of sarcopenia in elderly men but not in elderly women. More detailed prospective studies are needed.


**5-27**



**Comparison between the revised and the first version of the European Consensus for the diagnostic of sarcopenia in a follow‐up study of Chilean older people**



**Lydia Lera** and Carlos Márquez and Bárbara Angel and Rodrigo Saguez and Cecilia Albala


*INTA, University of Chile, Macul, Chile*



**Backgroud:** The care of older people requires early diagnosis of sarcopenia at primary care attention, therefore it is necessary to have the best diagnostic tool for its identification. The aim of this study is to compare the predicting validity of sarcopenia diagnosed with the first (EWGSOP1) and revised (EWGSOP2) versions of the European Consensus criteria, for functional limitations and falls in Chilean older people.


**Methods:** Follow‐up (median = 4.8 years) of 430 community‐dwelling participants (68.2 ± 4.8 years; 69.5% females) free of functional limitations, from 1006 subjects with measurements at baseline. The subjects were classified with sarcopenia by EWGSOP1 and EWGSOP2 criteria at baseline, to observe functional limitations and falls at the end of follow‐up. The participants had measurements of muscle mass by dual‐energy X‐ray absorptiometry, gait speed, and handgrip strength. Self‐reported and observed functional limitations, falls, and history of chronic diseases were also registered. χ^2^ test and logistic models were used for the analysis.


**Results**: At baseline the prevalence of sarcopenia was higher with EWGSOP1 than EWGSOP2 (19.1% vs. 10.5%). EWGSOP1 criteria classified 40 subjects (9.3%; 29 females) as sarcopenic that EWGSOP2 classified as nonsarcopenic (no difference by sex). At the end of follow‐up, after adjusting by age, sex, morbidity, nutritional state, and lean/fat ratio, sarcopenic subjects diagnosed by both criteria and sarcopenic subjects diagnosed by EWGSOP1 criteria and diagnosed as nonsarcopenic by EWGSOP2, presented risk of functional limitation (OR = 5.3; 95% CI: 1.4–19.4; OR = 3.2; 95% CI: 1.1–9.2) and of falling (OR = 3.8; 95% CI: 1.3–11.6; OR = 4.2; 95% CI: 1.8–10.0).


**Conclusions:** The prevalence of sarcopenia diagnosed by EWGSOP2 was significantly less than by EWGSOP1. The subjects that was classified as nonsarcopenic by EWGSOP2 but sarcopenic by EWGSOP1 showed a similar risk of functional limitation and of falling than sarcopenic subjects. The need to identify the majority of people at risk makes EWGSOP1 criterion very valuable yet.


**Funding**: Fondef IT15I10053, Fondecyt 1080589.


**5-28**



**Prevalence of sarcopenia in elderly patients admitted to a universitary hospital: a pilot study**



**Maria Cláudia Bernardes Spexoto**
^1^, Juliana Yukari Saganuma^2^ and Talita Yoshimura da Costa^2^



^1^
*Faculty of Health Sciences, Federal University of Grande Dourados (UFGD) and Tutora of Multiprofessional Health Residency Program in Cardiovascular Care, Mato Grosso do Sul, Brazil;*
^2^
*Resident Nutritionist of Multiprofessional Health Residency Program in Cardiovascular Care, Federal University of Grande Dourados (UFGD), Mato Grosso do Sul, Brazil*



**Background:** To assess the prevalence of sarcopenia and the associated factors is of great importance in order to describe and implement preventive actions in public hospitals. The objective was to estimate the prevalence of sarcopenia and its associated factors in hospitalized elderly patients.


**Methods:** Cross‐sectional pilot study with 39 hospitalized elderly patients (67,92 ± 8,80 years). Inclusion criteria were elderly patients of both sexes admitted to the surgical clinic of the Hospital of Federal University of Grande Dourados in the first 48 h. Patients with respiratory precaution, oedema or impossibility of hand evaluation, cognitive impairment, neurodegenerative diseases or severe psychiatric dysfunction, and the indigenous population were excluded. To assess the frequency of sarcopenia, the new criteria proposed by the European Working Group Sarcopenia in Older People (EWGSO). Calf circumference (CC) was used to assess muscle mass. This research is approved by the Research Ethics Committee/University (Protocol: 06426818.0.0000.5160). Descriptive statistics and the χ^2^ test were performed. It was considered 5% of significance.


**Results:** Most were male (53.8%), married (74.4%), not working (71.8%), economic class C* (69.2%) and had one to three chronic diseases (CD) (74.4%). The patients had handgrip strengths (HS) equal to 26.43 ± 9.19 kg/f (right) and 25.69 ± 10.15 kg/f (left). The CC was 34.88 ± 4.04 cm with 18.4% loss muscle mass. The average gait speed was 7.84 ± 7.74 s (4 meters). We obtained 78.1% without risk of sarcopenia (HS = 27.4 ± 9.33 kg/f), 15.6% with risk for sarcopenia (HS = 25.5 ± 8.06 kg/f), and 6.3% with severe sarcopenia (HS = 17,0 ± 7.07 kg/f). The frequency of sarcopenia was not associated with gender (*P* = .962), work activity (*P* = .568), presence of CD (*P* = .945) but was significantly related to body mass index (*P* = .021), CC (*P* = .003), and right handgrip strength (*P* = .030).


**Conclusions:** Sarcopenia is associated with nutritional status and patients hospitalized for surgery are already identified at risk for sarcopenia or severe sarcopenia. We continue with data collection to improve the analysis.

*Economic class C: Average income (home) equal to 572 EUR (Central Bank converter on July 26, 2019)


**5-31**



**Multiple biomarker approach to identify the patients with low muscle mass in heart failure**



**Masaaki Konishi**
^1,3^, Eiichi Akiyama^1^, Ryousuke Sato^1^, Yasushi Matsuzawa^1^, Kiyoshi Hibi^1^, Toshihiro Misumi^2^, Kouichi Tamura^3^ and Kazuo Kimura^1^



^1^
*Division of Cardiology, Yokohama City University Medical Center, Yokohama, Japan;*
^2^
*Department of Biostatistics, Yokohama City University School of Medicine, Yokohama, Japan;*
^3^
*Department of Medical Science and Cardiorenal Medicine, Yokohama City University Graduate School of Medicine, Yokohama, Japan*



**Background:** Identification of sarcopenia is still challenging in patients with heart failure (HF). Serum creatinine/serum cystatin C ratio (Cr/CyC) could reflect muscle mass, whereas B‐type natriuretic peptide (BNP) might have an interaction with skeletal muscle metabolism. We sought to assess the validity of several biomarkers as a predictor of muscle mass.


**Methods:** We measured body components using the dual‐energy X‐ray absorptiometry (DXA) in 207 hospitalized patients with HF (70 ± 13 years, 63% male, ejection fraction 38 ± 16%). DXA and BNP were measured in a stable condition after decongestion therapy.


**Results:** The average appendicular skeletal mass index (ASMI) was 6.80 ± 1.20 kg/m^2^ in men and 5.66 ± 1.05 kg/m^2^ in women and the prevalence of the patients with low ASMI defined by Asian Working Group for Sarcopenia was 53%. Cr/CyC was associated with body mass index (BMI: *r* = 0.20, *P* = 0.005) and ASMI (*r* = 0.39, *P* < 0.001) but not fat mass index (*r* = 0.02, *P* = 0.75) whereas Ln BNP with BMI (*r* = −0.32, *P* < 0.001), ASMI (*r* = −0.39, *P* < 0.001), and fat mass index (*r* = −0.23, *P* < 0.001). Multivariable linear regression analysis revealed that BMI (β = 0.64, *P* < 0.001), male sex (β = −0.34, *P* < 0.001), sodium (β = 0.08, *P* = 0.037), and Cr/CyC (β = 0.11, *P* = 0.011), but not Ln BNP (β = −0.06, *P* = 0.16) was independently associated with ASMI. An optimal cut‐off of BMI to identify those with low ASMI was 21.8 kg/m^2^ for men and 20.0 for women, which had a sensitivity of 78%, specificity of 90%, and accuracy of 83%, respectively. To add BNP (cut‐off: >400 pg/mL), but not Cr/CyC, after BMI raised sensitivity up to 90% without reducing accuracy (sensitivity 77% and accuracy 84%, respectively).


**Conclusions:** Cr/CyC was significantly associated with ASMI, independent of BMI. Using BNP after BMI, we could effectively identify patients with low ASMI in HF.


**5-32**



**Evaluation of sacrcopenia with bioimpedance variables and handgrip strength in middle‐aged Italian obese women**



**Enza Speranza** and Rosa Sammarco and Delia Morlino and Luisa Truocchio and Olivia Di Vincenzo and Iolanda Cioffi and Maurizio Marra and Fabrizio Pasanisi


*Department of Clinical Medicine and Surgery, Federico II University Hospital, Naples, Italy*



**Background:** Sarcopenic obesity (SO) is a condition where fat mass (FM) excess and muscle mass depletion coexist. A clear definition for SO is currently lacking, and there is therefore a need to develop a standardized approach of defining SO using body composition assessment. The aim of this study is to evaluate the prevalence of sarcopenic obesity in middle‐aged adults with obesity (BMI > 30) using BIA and handgrip strength as screening tools according to EWGSOP criteria.


**Methods**
*:* We studied 70 women [age 50 ± 8.2 years; weight 101.2 ± 19.1 kg, BMI 39.9 ± 7.27 kg/m^2^, fat‐free mass (FFM) 54.3 ± 8.9 kg, FAT 46.9 ± 12.9 kg, PFAT 45.8 ± 5.7%]. Antropometric measurements and bioimpedance analysis (BIA) at 50 kHz (DS Medica) were performed early in the morning; skeletal mass was calculated according to Janssen equation SM (kg) = (h^2^/BIA resistance*0.401) + (sex*3.825) + (age*0.071) + 5.102; where height (h) is in cm, BIA resistance is in ohms; male sex = 1 and female =0.The mean HGS was the average value of three handgrip measurements of the dominant hand. Sarcopenia was defined with two indexes: SMP INDEX = SM (kg)/body mass (kg) * 100 and SMI = SM/h^2^.


**Results:** Mean SM was 23.8 ± 3.90 kg, mean SMP was 23.9 ± 3.09, mean SMI was 9.41 ± 1.41, mean HGS was 20.4 ± 4.93 kg (more than 50% of patients had mean HGS < 20 kg). According to Janseen cut‐off of sarcopenia, we found that 31 subjects (44.3%) could be defined presarcopenic obese according to SMP index, whereas no one was defined sarcopenic according to SMI index. If we consider both SMP index and handgrip strength, 27.4% of the subjects were defined sarcopenic.


**Conclusions:** Sarcopenia rates vary widely based on different definitions. When SMP is used, we have observed the highest prevalence of Sarcopenia, whereas when we use also handgrip strength, we found lowest prevalence of sarcopenia. Further studies are required in a larger population to define SO.


**5-33**



**Characterization of molecular profile of sarcopenia in osteoporotic and osteoarthritic patients**



**Chiara Greggi**
^1,2^, Manuel Scimeca^3,4,5^, Virginia Veronica Visconti^1,2^, Matteo Primavera^1,2^, Laura Palmieri^1,2^, Riccardo Lundusi^1,2^, Elena Gasbarra^1,2^ and Umberto Tarantino^1,2^



^1^
*Department of Clinical Sciences and Translational Medicine, University of Rome Tor Vergata, Rome, Italy;*
^2^
*Department of Orthopedics and Traumatology, "Policlinico Tor Vergata" Foundation, Rome, Italy;*
^3^
*Department of Biomedicine and Prevention, University of Rome "Tor Vergata", Rome, Italy;*
^4^
*University of San Raffaele, Rome, Italy;*
^5^
*Fondazione Umberto Veronesi (FUV), Milano, Italy*



**Background:** Sarcopenia represents an important risk factor for osteoporosis. Indeed, sarcopenia might decrease bone strength by reducing mechanical loading: less time the skeleton is loaded due to relative immobility, thus bone formation is reduced. The aim of this study was to identify the main molecular pathways involved in physio‐pathogenesis of sarcopenia. In particular, we investigated the expression of BMP2, BMP4, BMP7, Myostatin, MDM2, and vitamin D receptor (VDR) and their relationship with muscle stem cells activity (PAX‐7, myogenin, CD44).


**Methods:** We enrolled 32 patients that underwent hip arthroplasty for femoral fracture (OP), and 68 patients underwent hip arthroplasty for osteoarthritis. The main clinical/anamnestic data as well as haematochemical values and instrumental parameters were collected. Serial paraffin sections were used for morphometric and immunohistochemical analysis.


**Results:** Clinical and instrumental evaluation allowed us to characterize patients enrolled in this study. OP group included 25 patients with fragility hip fracture (T‐score ≤ −2.5 SD); whereas OA group included 25 patients with positive radiogram for hip OA (T‐score ≥ −2.5 SD). Morphometric investigations demonstrated the delay in the onset of sarcopenia in OA patients. Immunohistochemical analysis showed that BMPs and nuclear VDR were more expressed in OA patients than OP. As concern myostatin, we noted a strongly association between its expression and degenerative phenomena observed in muscle biopsies of OP patients. Also, for the first time, we correlated the expression of MDM‐2 with muscle degeneration. Lastly, analysis of PAX‐7, myogenin, and CD44 allowed us to observe an increase of satellite cells activity in OA patients compared to OP.


**Conclusions:** The identification of the molecular profile of sarcopenia can provide the rational for new therapies. In particular, our data allowed us to propose the use of human recombinant BMPs, VIT.D3 supplementation, and the anti‐myostatin molecules as drugs capable to prevent or treat the sarcopenia of OP patients.


**5-34**



**Predictor of sarcopenia and its association with disease activity status and physical function in rheumatoid arthritis**



**Ricardo M. Xavier**
^1,2^, Rafaela C.E. Santo^1,2^, Jordana Miranda de Souza Silva^1,2^, Joshua Baker^3^, Vanessa Hax^1,2^, Claiton Viegas Brenol^1,2^, Lidiane I. Filippin^4^ and Priscila Lora^5^



^1^
*Universidade Federal do Rio Grande do Sul, Porto Alegre, Brazil;*
^2^
*Serviço de Reumatologia do Hospital de Clínicas de Porto Alegre, Porto Alegre, Brazil;*
^3^
*University of Pennsylvania, Philadelphia, USA;*
^4^
*Universidade La Salle, Canoas, Brazil;*
^5^
*Universidade do Vale do Rio dos Sinos, São Leopoldo, Brazil*



**Background:** Rheumatoid arthritis (RA) is characterized by chronic inflammation, leading to joint damage. RA patients also present altered body composition, including loss of appendicular lean mass index (ALMI) with preservation or increase in fat mass index (FMI), but very little is known on the prevalence, progression, and impact on function of this complication. The adiposity is a confounder that may mask relationships between physical functioning and ALMI. This prospectively evaluate muscle mass relative to fat mass (ALMI_FMI_) and its associations with disease activity status, physical function, and use of biologic therapies.


**Methods**: RA patients were recruited and followed for 12 months. ALMI and FMI were assessed by DEXA. RA patients were divided by remission or non‐remission and use of biologic therapy. Physical function was assessed by HAQ‐DI. Pearson correlations and GEE analyses were used (*P* < 0.05).


**Results:** Of the 90 patients analysed, most were women (86.7%), with mean age of 56.5 ± 7.3 and long disease duration. At baseline, the most patient showed remission disease activity, 30% of the patients were treated with biologic therapy, and after 12 months, these parameters were stable. twelve percent of RA patients showed low ALMI_FMI_ (z‐score ≤ −1) at baseline and 16% after 12 months. After 12 months, ALMI_FMI_ was inversely associated with HAQ‐DI (*r* = −0.3; *P* < 0.05). Women in remission showed higher ALMI_FMI_ in both times (*P* < 0.05). The use of biologic therapy was not related to changes in ALMI_FMI_.


**Conclusions:** Changes in body composition were observed after only 12 months in RA patients with stable disease. Disease activity status was associated with changes on ALMI_FMI_. Low ALMI_FMI_ was associated with decreased physical function in RA patients. Further long‐term follow‐up studies are necessary to elucidate the risk factors, impacts, and strategies to alleviate muscle loss in these patients.


**5-35**



**Myopenia, myosteatosis, and systemic inflammation are dependent on ethnicity in patients undergoing surgery for colorectal cancer**



**Edward Tobias Pring**
^1,2,3,4^, Laura E. Gould^1,2^, Georgios Malietzis^1,3^, Thanos Atanasiou^1,3^ and John T. Jenkins^1,2,3^



^1^
*George Davies Research Fellowship, Leicester, UK;*
^2^
*BiCyCLE Research Group, St. Mark's Hospital, Harrow, UK;*
^3^
*Department of Surgery, St. Mark's Hospital, Harrow, UK;*
^4^
*Imperial College London, London, UK*



**Background:** Heterogeneity of body composition (BC) exists across ethnic groups. We know that in colorectal cancer (CRC) patients, myopenia and myosteatosis are related to a systemic inflammatory state and poorer outcomes. Despite this difference in BC between ethnic groups, no studies have examined the relationship between ethnicity, muscle mass, and inflammation in the presence of cancer. We aimed to examine this relationship with a view to elucidating a need to adjust existing cut‐off values.


**Method:** A prospectively maintained database of primary CRC patients undergoing surgery at a single UK specialist bowel hospital was analysed. Patient self‐declared ethnicity was coded using the NHS Data Dictionary codes from the Office of National Statistics 2001 UK census. These categories were further simplified for analysis into the broader categories listed within the NHS Data Dictionary. Preoperative computer tomographic BC analysis was performed using SliceOmatic v5.0 and ABACS L3. Cut‐off values for BC variables were used from previous work by Prado *et al* (2008) and Doyle *et al* (2013).


**Results:** A total of 1401 patients were included in the analysis; ethnicity data was held for 1098 individuals. Sixty‐eight percent of patients were white, 19% Asian/Asian British, and 8% Black/Black British, the remainder were of mixed race or “other ethnic group.” Black patients were significantly less myopenic than white and Asian patients *P* = 0.0005. White patients were significantly more myosteatotic than Asian patients *P* = 0.0005. Ethnicity was associated with a clinically significant difference in preoperative neutrophil‐to‐lymphocyte ratio (NLR > 3) *P* = 0.0005 but no clinically significant relationship between platelet to lymphocyte ratio (PLR > 130) *P* = 0.419.


**Conclusions:** In myopenia and myosteatosis, there are significant differences that exist between ethnic groups; this is also reflected in a significant difference in systemic inflammation. This in turn may have a bearing on outcomes from colorectal cancer. Further investigation is warranted to define cut‐off values further taking ethnicity into account.


**5-36**



**Loss of skeletal muscle mass during neoadjuvant chemotherapy and the relation to survival in patients with ovarian cancer: a prospective analysis of the OVHIPEC‐1 cohort**



**Jorne Ubachs**
^1,2,3,4^, Simone Koole^5,6^, Leigh Bruijs^5^, Max Lahaye^7^, Cristina Fabris^8^, Jules Schagen van Leeuwen^9^, Henk Schreuder^10^, Ralph H. Hermans^11^, Ignace H. de Hingh^12^, Jacobus van der Velden^13^, Henriette J. Arts^14^, Leon Massuger^15^, Jacco Bastings^1^, Roy F.P.M. Kruitwagen^1,2^, Sandrina Lambrechts^1,2^, Steven W.M. Olde Damink^3,4,16^, Sander S. Rensen^3,4^, Toon Van Gorp^17^, Gabe Sonke^6^ and Willemien van Driel^5^



^1^
*Department of Obstetrics and Gynecology, Maastricht University Medical Centre, Maastricht, The Netherlands;*
^2^
*GROW ‐ School for Oncology and Developmental Biology, Maastricht University, Maastricht, The Netherlands;*
^3^
*Department of Surgery, Maastricht University Medical Centre, Maastricht, The Netherlands;*
^4^
*NUTRIM, School of Nutrition and Translational Research in Metabolism, Maastricht University, Maastricht, The Netherlands;*
^5^
*Department of Gynecology, The Netherlands Cancer Institute, Amsterdam, The Netherlands;*
^6^
*Department of Medical Oncology, The Netherlands Cancer Institute, Amsterdam, The Netherlands;*
^7^
*Department of Radiology, The Netherlands Cancer Institute, Amsterdam, The Netherlands;*
^8^
*Department of Radiology, University of Verona Hospital, Verona, Italy;*
^9^
*Department of Obstetrics & Gynecology, Sint Antonius Hospital, Nieuwegein, The Netherlands;*
^10^
*Department of Gynecological Oncology, UMC Utrecht Cancer Center, Utrecht, The Netherlands;*
^11^
*Department of Gynecology and Obstetrics, Catharina Hospital, Eindhoven, The Netherlands;*
^12^
*Department of Surgery, Catharina Hospital, Eindhoven, The Netherlands;*
^13^
*Department of Obstetrics and Gynecology, Academic Medical Center, Amsterdam, The Netherlands;*
^14^
*Department of Gynecological Oncology, University Medical Center Groningen, Groningen, The Netherlands;*
^15^
*Department of Gynecological oncology, Radboud University Medical Center, Nijmegen, The Netherlands;*
^16^
*Department of Visceral‐ and Transplantation Surgery, RWTH Aachen University, Aachen, Germany;*
^17^
*Department of Obstetrics and Gynecology, Division of Gynecological Oncology, University Hospitals Leuven, Leuven Cancer Institute, Leuven, Belgium*


Jorne Ubachs and Simone Koole equally contributed to this article.


**Background:** Skeletal muscle depletion in women with advanced ovarian cancer has been associated with adverse clinical outcome and survival. To validate earlier results in a homogenous population, we analysed whether a decrease in skeletal muscle index (SMI) during neoadjuvant chemotherapy (NACT) is associated with worse outcome in patients with stage III epithelial ovarian cancer, who were included in the OVHIPEC trial.


**Methods:** Within the phase III OVHIPEC trial, 245 patients with stage III ovarian cancer were randomized after three cycles of NACT with carboplatin and paclitaxel to receive interval cytoreductive surgery (CRS) with or without HIPEC. Randomization was performed after at least stable disease after two cycles of NACT and when complete or optimal CRS was achieved. CT scans performed at baseline (Timepoint 1), and after two cycles of NACT (Timepoint 2) were selected. A slide on the third lumbar level was selected from each CT scan, and the difference in SMI between both scans (ΔSMI) was calculated using SliceOMatic. Overall and recurrence‐free survival of patients with a decrease or increase in ΔSMI were performed using Kaplan–Meier estimates and log‐rank tests.


**Results:** Of the 245 patients randomized in the OVHIPEC trial, SMI and ΔSMI of scans at both timepoints were available for 212 patients (87%). After a median follow‐up of 4.7 years, 116 of 212 patients (55%) had died. In survival analysis, 43 of 74 patients (58%) in the group with a decrease in ΔSMI, and 73 of 138 of the patients (53%) in the group with stable/increase in ΔSMI had died. Median overall survival did not differ significantly (*P* = 0.764).


**Conclusions:** A decreasing skeletal muscle index during neoadjuvant chemotherapy was not associated with worse outcome in patients with stage III ovarian cancer, who were treated with complete/optimal interval CRS and six cycles of chemotherapy within the OVHIPEC trial.


**5-38**



**Agreement between measurements of body composition by dual‐energy X‐ray absorptiometry and bioimpedance analysis**


Tania Garfias Macedo^1,2^, Mirela Vatic^1,2^, Guilherme Wesley Peixoto da Fosenca^1,2,3^, Stefan Anker^4,6^, Wolfram Doehner^4,5,6^, Stephan von Haehling^1,2^ and **Nicole Ebner**
^1,2^



^1^
*Department of Cardiology and Pneumology, University Medical Center Goettingen, Goettingen, Germany;*
^2^
*German Centre for Cardiovascular Research (DZHK) partner site Goettingen, Goettingen, Germany;*
^3^
*Heart Institute, University of São Paulo Medical School, São Paulo, Brazil;*
^4^
*Berlin Brandenburg Center for Regenerative Therapy, Charité ‐ University Medical School, Berlin, Germany;*
^5^
*Department of Cardiology (Virchow Klinikum), Charité Universitätsmedizin Berlin, Berlin, Germany;*
^6^
*German Centre for Cardiovascular Research (DZHK) partner site Berlin, Berlin, Germany*



**Background:** Sarcopenia is getting increased awareness as a relevant comorbidity in patients with chronic heart failure (HF). Its impact in quality of life and mortality increases the importance of having valid measurement methods available for all patients, despite their condition or device implantation. The aim of this study is to compare the measurements of body composition on patients and controls and to compare the classification of patients with and without sarcopenia with respect to the given methods.


**Methods:** A total of 130 ambulatory patients with stabile chronic HF [age: 68 ± 10.13 years; NYHA(I/II/III/IV):13/73/39/1; BMI: 29.81 ± 5.3 kg/m^2^; Female: 39 (30%)] were enrolled as part of the Studies Investigating Co‐morbidities Aggravating Heart Failure (SICA‐HF). Additionally, 42 healthy controls [age: 64 ± 10.8 years; BMI: 25.29 ± 3.32 kg/m^2^; Female: 20 (48%)] were enrolled. Body composition on both, patients and controls, was measured using dual‐energy X‐ray absorptiometry (DEXA) and bioimpedance analysis (BIA). Depending on the method to measure muscle mass, we defined sarcopenia as the appendicular muscle mass index (ASMI) <7.26 for males or <5.45 for females from the DEXA scan and as the skeletal muscle index (SMI) ≤8.50 for males or ≤5.75 for females from the BIA scan. The ASMI was defined as the lean mass (kg) of both arms and legs combined (ASM) and then normalized by height squared (m^2^). Accordingly, using the resistance from BIA, SMI was calculated as absolute skeletal muscle mass (kg) normalized by height squared (m^2^).


**Results:** Overall, patients and controls were similar in age (*P* = 0.06) and showed similar composition of appendicular muscle mass (23.44 ± 5.19 vs. 22.59 ± 5.51 kg, *P* = 0.37). Patients, in contrast to controls, presented with higher BMI (*P* < 0.001), total fat mass, and total lean mass (30.72 ± 10.46 vs. 21.58 ± 7.64 kg, *P* < 0.001 and 54.87 ± 11.57 vs. 49.81 ± 10.78 kg, *P* = 0.01, respectively). In comparison to the DEXA measurements, we also obtained for fat mass (FM), fat‐free mass (FFM), and skeletal muscle mass by BIA a difference between patients and controls (29.35 ± 9.92 vs. 22.08 ± 7.07 kg, *P* < 0.001; 58.89 ± 12.84 vs. 51.82 ± 11.73 kg, *P* < 0.001; 26.85 ± 6.4 vs. 24.35 ± 6.42 kg, *P* = 0.04, respectively).

In order to show the compatibility of the two methods, we calculated the limits of agreement based on the Bland–Altman plot. Comparing fat mass by DEXA with fat mass by BIA, we obtained a mean of the differences with limits of agreement lower and upper as (1.36 [−7.86; 10.59]) on patients and as (−0.51 [−7.85; 6.83]) on controls. The comparison of lean mass (DEXA) with fat‐free mass (BIA) was given on patients by (−2.02 [−9.83; 5.79]) and on controls (−4.02 [−12.7; 4.65]). Comparing the definitions for sarcopenia as described above, we identified 13 (10%) patients with sarcopenia using the cut‐off for ASMI by DEXA and 24 (19%) patients with sarcopenia using SMI by BIA. Restricted to these patients, we calculated the Bland–Altman plot, obtaining the following mean of differences with limits of agreement. For the patients with sarcopenia defined by DEXA cut‐offs, the mean of differences for the fat‐free mass was (−6.5 [−15.56; 2.56]). In comparison to this result, the patients with sarcopenia defined by BIA cut‐offs, the means of the differences was (−2.91 [−12.75; 6.92]).


**Conclusions:** We have shown that the measurements of fat‐free mass BIA and lean mass DEXA are comparable. The definitions for sarcopenia using the respective cut‐offs deliver similar persistence on this population. Moreover, in order to increase the accordance in the persistence given by the different definitions, we encourage validating the comparison on a larger population.


**6-21**



**Characterization of central and peripheral age‐related changes in the neuromuscular system of C57BL/6J mice**


Alba Blasco^1^, Sílvia Gras^1^, Olga Tarabal^1^, Lídia Piedrafita^1^, Anna Casanovas^1^, Guillem Mòdol^2^, Xavier Navarro^2^, Josep Esquerda^1^, **Alejandro Barranco**
^3^, Tapas Das^4^, Suzette L. Pereira^4^, Ricardo Rueda^3^ and Jordi Calderó^1^



^1^
*Unitat de Patologia Neuromuscular Experimental, Dept. de Medicina Experimental, IRBLLEIDA, Universitat de Lleida, Lleida, Spain;*
^2^
*Dept. de Biologia Cel·lular, Fisiologia i Immunologia, Institut de Neurociències, Universitat Autònoma de Barcelona, Barcelona, Spain;*
^3^
*Abbott Nutrition, Strategic Research, Granada, Spain;*
^4^
*Abbott Nutrition, Strategic Research, Columbus, OH, USA*


*First Co‐authors

Ageing is associated with a reduction of muscle mass and strength, a process called sarcopenia. Structural and molecular changes in the cellular elements that shape the neuromuscular system could be causative factors of the age‐related skeletal muscle involution. Therefore, interventions aimed at preventing these changes may have a great impact in the preservation of skeletal muscle function in elderly. To gain new insights in the alterations occurring in the neuromuscular system with age, motor behavioural and electrophysiological tests and histological and immunocytochemical analyses were performed in young adult and old C57BL/6J mice. We found that although old mice did not exhibit significant changes in the size of spinal cord motoneurons (MNs), these displayed a marked loss of cholinergic and proprioceptive inputs. Ageing also induced prominent astrogliosis and microgliosis around MNs, with spinal cord of old animals exhibiting a significant increase in the density of pro‐inflammatory M1 microglia and A1 astroglia. Compared to muscles from young adult mice, those from old animals exhibited higher numbers of both denervated and polyinnervated neuromuscular junctions (NMJs), a higher proportion of myofibers showing increased size and central nuclei, and augmented expression of different molecules related to NMJ stabilization and plasticity including CGRP, GAP‐43, FGBP1, and TGF‐β1. These changes were found to be more prominent in *soleus* and *gracilis* than in EDL and tibialis anterior muscles. In relation to young adult mice, old animals had a significant reduction in the nerve conduction velocity and a decline in the amplitude of the compound muscle action potential in distal plantar muscles. Further work is necessary to study the relevance of these structural and molecular changes and their impact on motor activity defects linked to ageing.


**6-22**



**The evaluation of body composition and lifestyle habits in a healthy population**



**Renata Gonçalves Pinheiro** and Thaísa Hoffmann Jonasson and Tatiana Munhoz da Rocha Lemos Costa and Victoria Zeghbi Cochenski Borba


*Serviço de Endocrinologia ne Metabologia do Hospital de Clínicas da Universidade Federal do Paraná (SEMPR), Curitiba, Brazil*



**Background:** Changes in body composition (BC) mainly reduction in lean muscle mass affect functional capacity. The objective of this study is to evaluate BC and correlate it with anthropometric, laboratory, and clinical parameters [nutritional, comorbidities, bone mineral density (BMD)].


**Methods:** Observational, cross‐sectional study that included healthy men and women ≥18 years old with body mass index (BMI) ≥18.5–29.9 kg/m^2^. Athletes, smokers, those with disease, or in use of drugs that could affect BC were excluded. Patients answered questionnaires about socio‐demographics, food intake, and physical activity [“*International Physical Activity Questionnaire*” (IPAQ)] data. *Short Physical Performance Battery* (SPPB), total body densitometry exam by DXA, and laboratory test were performed.


**Results:** From 1100 individuals invited, 299 were included, 150 men (45.1 ± 20.4 years) and 149 women (47.1 ± 19.4 years), *P* = 0.45. There was a negative correlation between % total fat (TF) and age in men and women (*R* = 0.5, *P* < 0.00, for both) mainly in trunk, superior members, and android region. Men showed lower %TF accordingly to their physical activity (*P* < 0.005); however, women did not. Vitamin D and calcium ingestion were negatively correlated to android fat (AF). Multivariate analysis showed that %TF was dependent of age, BMI, abdominal circumference (AC), and gender (male); however, when the level of physical activity was included in the model, age lost significance when individuals were active (R2 = 0.75, *P* < 0.0001). Lean body mass (LBM) was higher accordingly to the level of physical activity in men (*P* < 0.002), but in women, although high LBM was observed between the active individuals compared to the sedentary, no difference was seen between the active and insufficiently active. In multivariate analysis, LBM was dependent of BMI, AC, male gender, and active by IPAQ (*P* < 0.005). AF was higher in individuals with comorbidities (*P* < 0.005).


**Conclusions:** Physical activity is an independent factor influencing body composition in men and women, regardless of age.


**6-24**



**Low muscle mass as a predictor of mortality risk among older adults: a systematic review**


Rômulo Roosevelt da Silva Filho and Cristina Camargo Pereira and **Erika Aparecida Silveira**



*Universidade Federal de Goiás, Goiania, Brazil*



**Background:** Sarcopenia is a geriatric syndrome, and it is associated with poor health outcomes such as death. We aimed to review the evidence of low muscle mass (LMM) as a predictor of mortality among older adults.


**Methods:** Systematic review of observational cohort studies was conducted according to the PRISMA guidelines. PubMed and Scielo databases were searched. The search strategy used was (elderly OR “older adults”) and (community OR noninstitutionalized) and “low muscle mass” and mortality. Inclusion criteria were (i) prospective cohort studies; (ii) studies investigating whether sarcopenia according to LMM was a predictor of mortality; (iii) studies published in Spanish, Portuguese, and English languages; and (iv) studies published within the last 5 years. Exclusion criteria were (i) type of participants: hospitalized and (ii) article type: review articles, letters, dissertation, and thesis.


**Results:** Of 17 studies identified, three were included. Studies were conducted in Australia and Brazil. Follow‐up periods varying from 4 to 10 years, and the relative risk (RR), odds ratio (OR), or hazard ratio (HR) were used. The participant's minimum age ranged from 50 to 70 years old. All studies used dual‐energy X‐ray absorptiometry (DXA) as diagnostic criteria for muscle mass. The prevalence of sarcopenia ranged from 20.73% to 26.63% among men and 21.33% to 21.51% among women. LMM was significantly associated with mortality risk (RR = 1.54, 95% CI: 1.14–2.08, *P* < 0.05). Analysis by sex demonstrated that the mortality risk was higher among women (OR = 62.88, 95% CI: 22.59–175.00, *P* < 0.001) than men (OR = 11.36, 95% CI: 2.21–58.37, *P* = 0.004). However, there was a study that not found mortality risk among men (HR = 0.88, 95% CI: 0.70–1.11, *P* = 0.29). Association between LMM and mortality risk was not found when predefined cut‐offs were used to diagnosis muscle mass.


**Conclusions:** Sarcopenia according to LMM increases mortality risk among noninstitutionalized older adults. However, further researches are needed to explain gender discrepancies.


**6-25**



**Correlation among sarcopenia stages and fear of falling in prefrail community‐dwelling older women**



**Renata Gonçalves Pinheiro Correa** and Gabriela Tormes and Jarbas Melo Filho and Audrin Said Vojciechowski and Simone Biesek and Victoria Zeghbi Cochenski Borba and Anna Raquel Silveira Gomes


*Universidade Federal do Paraná, Pinhais, Brazil*



**Background:** Sarcopenia is characterized by a reduction in muscle mass, muscle strength, physical performance and can contribute to falls. This study's purpose is to correlate the stages of sarcopenia and fear of falling in prefrail community‐dwelling older women.


**Methods:** Cross‐sectional study with 70 prefrail elderly women (71.2 ± 4.5 years; BMI: 9.4 ± 4.1 kg/m), classified according to the Fried frailty phenotype. Handgrip strength (HGS) through dynamometer; muscle mass (MM) was estimated by calf circumference (CC) and Lee equation (MM‐Lee); gait speed in 4 meters (GS 4 m); lower limb strength (chair‐stand Test ‐ CST5rep); fear of falling (*Falls efficacy Scale – Internacional* FES‐I BRAZIL).


**Results:** The older women showed 20.0 ± 6.0 kgf (HGS), CC 36.4 ± 3.7 cm, and GS4m 1.02 ± 0.2 m/s. It was detected nonsarcopenia in 80% (*n* = 59) (CST5rep = 11.6 ± 3.3 s; MM ‐ Lee = 7.9 ± 0.9 kg/m^2^; FES‐*I* = 22.5 ± 5.6), probable sarcopenia in 15.7% (*n* = 11) (TSL5X = 11.9 ± 3.9 s; MM ‐ Lee = 7.8 ± 1.7 kg/m^2^; FES‐*I* = 28.3 ± 8, 3 cm), and sarcopenia in 4.3% (*n* = 3) (TSL5X = 11.9 ± 3.4 s; MM ‐ Lee = 7.4 ± 0.7 kg/m^2^; FES‐*I* = 28.4 ± 6.8 cm) of participants. It was found the following correlations: nonsarcopenia and fear of falling (*P* = 0.357; *P* = 0.003) and probable sarcopenia and fear of falling (*P* = 0.309; *P* = 0.01).


**Conclusions:** The prefrail community‐dwelling older women in a probable sarcopenia stage and those with sarcopenia presented fear of falling indicating sporadic falls. However, even the nonsarcopenics showed association with fear of falling.


**6-26**



**Body mass index and mortality among community‐dwelling older adults from Southern Brazil**


Andressa Souza Cardoso^1^, Mariana Otero Xavier^2^, Caroline dos Santos Costa^2^, Elaine Tomasi^2^, Juraci Almeida Cesar^3^, Maria Cristina Gonzalez^1,2,4^, Thiago Gonzalez Barbosa e Silva^2^ and **Renata Moraes Bielemann**
^1,2^



^1^
*Post‐Graduate Program in Nutrition and Foods, Federal University of Pelotas, Pelotas, Brazil;*
^2^
*Post‐Graduate Program in Epidemiology, Federal University of Pelotas, Pelotas, Brazil;*
^3^
*Post‐Graduate Program in Public Health, Federal University of Rio Grande, Rio Grande, Brazil;*
^4^
*Post‐Graduate Program in Health and Behavior, Catholic University of Pelotas, Pelotas, Brazil*



**Background:** The objective of this study is to evaluate the association between body mass index (BMI) and mortality among community‐dwelling older adults in Pelotas, Brazil, using two different criteria to classify the BMI and considering the myopenia.


**Methods:** It is a cohort study started in 2014 with older adults living in the city of Pelotas, Southern Brazil. BMI was classified according to the World Health Organization (WHO) and Lipschitz. Deaths were identified up to April 2017. Calf circumference ≤33 cm in women and ≤34 cm in men diagnosed myopenia. Cox proportional hazards regression investigated the associations adjusting for sociodemographic and behavioural characteristics in addition to the number of chronic diseases.


**Results:** We interviewed 1451 older adults in 2014. Around 10% (*N* = 145) of the participants died in almost 3 years. Relationship between BMI and risk of mortality was L‐shaped. Participants with low weight had higher risk of mortaity in comparison to those with adequate BMI in both criteria. According to WHO criterion, overweight individuals had lower risk of mortality (HR: 0.58; 95% CI: 0.38; 0.87) in relation to those with adequate BMI. There was no statistical significance in the risk of mortality in overweight older adults using the Lipschitz criterion. Among participants with myopenia, hazard ratio for risk of mortality was below the null value in the overweight group in both criteria, although not statiscally significant, whereas those classified with low BMI according to Lipschitz had higher risk of mortality than participants with adequate BMI (HR: 2.09; 95% CI: 1.06; 4.14).


**Conclusions:** Low BMI increased the risk of mortality in up to a 3 year period among community‐dwelling older adults. The Lipschitz criterion seemed to be more adequate to identify high risk of mortality based on BMI in this sample. Higher BMI decreased the risk of mortality when myopenia was not taken into account.


**6-27**



**Calf circumference as a predictor of all‐cause mortality among noninstitutionalized elderly in Brazil**



**Cristina Camargo Pereira**
^1^, Valéria Pagotto^2^, Annelisa Silva e Alves de Carvalho Santos^3^ and Erika Aparecida Silveira^1^



^1^
*Health Sciences Graduate Program, School of Medicine, Universidade Federal de Goiás, Goiânia, Brazil;*
^2^
*Nursing Graduate Program, Universidade Federal de Goiás, Goiânia, Brazil;*
^3^
*Faculdade Unida de Campinas, Goiânia, Brazil*



**Background:** Calf circumference (CC) reflects muscle mass status in the elderly population. However, its effectiveness in predicting long‐term mortality risk has not been fully elucidated. We aimed to evaluate the effectiveness of CC in predicting mortality risk in noninstitutionalized elderly.


**Methods:** This is a 10 year follow‐up cohort study (Elderly Project/Goiania) with noninstitutionalized elderly individuals aged greater or equal 60 years. Trained nutritionists performed CC using a standardized procedure. Mortality data were collected from the Brazilian Mortality Information System of the Health Ministry. The association of mortality risk with low CC (<34 cm for men and <33 cm for women) was assessed by Cox regression. Statistical significance was set at *P* value < 0.05.


**Results:** We evaluated 416 elderly individuals with a mean age of 70.7 ± 7.1 years, and 66.1% were women. Mean CC was 34.7 ± 3.1 cm in men and 34.1 ± 3.6 cm in women, without statistical difference (*P* = 0.14). Prevalence of low CC was 36.3% (95% CI: 31.66–40.94), being 39.7% (95% CI: 31.54–47.89) in men and 34.6% (95% CI: 28.89–40.20%) in women, without statistical difference (*P* = 0.30). There were 144 deaths within an 8.5 year follow‐up period, representing 34.6% of the study sample. Cox regression analyses showed that mortality risk is greater in elderly with low CC (HR = 1.88 95% CI: 1.35–2.61) (*P* < 0.01) (Figure 1). Greater mortality risk was also observed among women (HR = 1.94 95% CI: 1.27–2.96) (*P* < 0.01) compared to men (HR = 1.74 95% CI: 1.03–2.96) (*P* = 0.04).

**Figure 1 jcsm12551-subcmp-0025-fig-0002:**
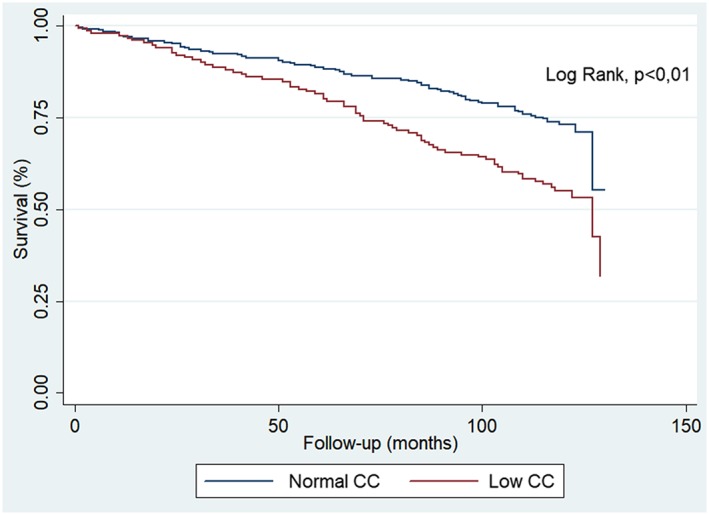
Survival curves of the study population according to calf circumference (CC). Low CC: <34 cm for men and < 33 cm for women.


**Conclusions:** Low CC is an anthropometric indicator with relevant impact on all‐cause mortality in both sexes of noninstitutionalized Brazilian elderly. Considering that CC is a non‐invasive, low‐cost, and easily standardized measure compared to gold standard methods, effort should be made to increase the use of this indicator in clinical practice.


**6-28**



**Dysmobility syndrome is associated with cognitive and functional impairment in middle‐aged and older adults of Mexico City**


Claudia Szlejf^1,2^, Lorena Parra‐Rodríguez^1^, **Miriam López‐Teros**
^3^ and Oscar Rosas‐Carrasco^1^



^1^
*Instituto Nacional de Geriatría, Mexico City, Mexico;*
^2^
*Center for Clinical and Epidemiological Research, Hospital Universitário, University of Sao Paulo, Sao Paulo, Brazil;*
^3^
*Universidad Iberomaericana, Mexico City, Mexico*



**Background:** Dysmobility syndrome is a new concept in the literature that may have a role as predictor of adverse outcomes in older people. The results of this article are relevant for the geriatric and gerontological scientific communities as they call attention for the association of two frequent geriatric syndromes with dysmobility syndrome: disability and cognitive impairment. The objective of this study is to investigate the frequency of dysmobility syndrome (DS) and its association with geriatric syndromes in community‐dwelling middle‐aged and older adults of Mexico City.


**Methods:** In this cross‐sectional analysis of the FraDySMex study, 534 participants aged 50 years and older were included. Body composition, gait speed, and grip strength were evaluated with DEXA, GAITRite instrumented walkway, and hand dynamometer, respectively. Sociodemographic characteristics, clinical history, falls, mental, nutritional, and functional status.


**Results:** Participants' mean age was 71.3 ± 9.5 years, 80.1% females. The frequency of DS was 28.1% (*n* = 150). After adjustment, geriatric syndromes associated with DS were cognitive impairment (OR = 2.38, 95% CI = 1.29–4.40, *P* = 0.006) and dependence in basic activities of daily living (OR = 2.21, 95% CI = 1.08–4.54, *P* = 0.031).


**Conclusions:** DS is independently associated with cognitive impairment and disability in community‐dwelling middle‐aged and older adults. Further studies are needed to better understand the role of DS as a geriatric syndrome predictor.


**6-29**



**Impact and risk factors for clinically relevant surgery‐related muscle loss in patients after major abdominal cancer surgery: study protocol and preliminary results from a prospective observational cohort study (MUSCLE POWER)**



**Judith E.K.R. Hentzen**, Laura van Wijk, Carlijn I. Buis, Alani R. Viddeleer, Geertruida H. de Bock, Cees P. van der Schans, Schelto Kruijff and Joost M. Klaase


*University Medical Center Groningen, Griningen, The Netherlands*



**Background:** Surgery‐related muscle loss (SRML) occurs in at least one out of three cancer patients within 1 week after major surgery. Though, this important phenomenon has hardly been investigated.


**Methods:** The MUSCLE POWER is a prospective, observational cohort study that investigates the presence, impact, and predictors for clinically relevant SRML in 178 cancer patients after major abdominal surgery using ultrasound measurements, squeeze and force measurements, and quality of life (QoL) questionnaires (Figure 1). Primary end point is the proportion of patients with clinically relevant SRML defined as ≥5% muscle loss within 1 week after surgery, measured by the anterior–posterior diameter of three different muscles: m. biceps brachii, m. rectus femoris, and m. vastus intermedius. Possible correlation with QoL and fatigue up to 6 months after surgery will be explored. Physical activity and protein intake during hospital stay will be monitored with a motility tracker and a nutrition dairy. Possible predictors for clinically relevant SRML—consisting of age ≥ 65 years, preoperative diabetes, preoperative sarcopenia, major postoperative complications (Clavien–Dindo ≥III), insufficient physical activity, and insufficient protein intake—will be investigated with a multivariable logistic regression analyses with a backward stepwise approach. Variables with *P* < 0.05 will be retrained in the final multivariable model.


**Results:** Preliminary results of the first 60 patients can be presented at the *Cachexia Conference 2019* in Berlin. We hypothesized that 50% of our patient population will have clinically relevant SRML which will lead to a reduced QoL and fatigue up to 6 months after surgery. We expect that the possible predictors investigated in this study can predict clinically relevant SRML prior to surgery.


**Conclusions:** The MUSCLE POWER study investigates the presence, impact, and predictors for clinically relevant SRML in cancer patients after major abdominal surgery. Crucial information to design future intervention studies to prevent postoperative muscle loss and improve postoperative outcome.

**Figure 1 jcsm12551-subcmp-0041-fig-0003:**
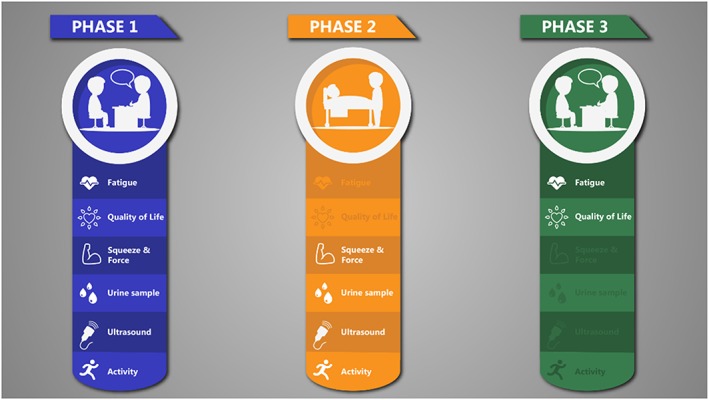
Study design. Phase 1: One day prior to surgery; Phase 2: Weeks after surgery till discharge; and Phase 3: Three and six months after surgery.


**6-30**



**“Dry” appendicular muscle mass as measured by segmental impedance spectroscopy during the 12 channel routine electrocardiogram relates to aortic dysfunction in chronic heart failure**



**Falko Skrabal**
^1,2^, Johannes Boyer^1,2^, Hasibullah Ehsas^1,2^ and Katharina Skrabal^1^



^1^
*Institute of Cardiovascular and Metabolic Medicine, Graz, Austria;*
^2^
*Medizinische Universität Graz, Graz, Austria*



**Background:** There are known relationships between sarcopenia, visceral fat, and arterial stiffness. We have thought to improve these relationships by correcting appendicular muscle mass measurements for excess extracellular water (“dry AppMM”) and to include all necessary measurements in the routine 12 channel ECG.


**Methods:** A 12 channel ECG supplies impedance measurements at multiple frequencies in six body segments (thorax, abdomen, and the extremities) and also impedance plethysmographic measurements at the four extremities [Medical Engineering & Physics 36 (2014) 896& 44(2017) 44]. All measurements are performed automatically without time delay during the routine ECG. AppMM, extracellular water (ECF), total body fat, and trunk fat were determined from segmental impedances at six body segments. DXA‐derived AppMM was also measured. From the peripheral impedance plethysmographic measurements, aortic volume wave velocity (aoVWV) was derived in analogy to carotid femoral volume wave velocity measured by mechanical transducers. “Dry AppMM” corrected for ECF excess, body fat and aoVWV were measured in 158 normotensive, healthy participants (72 males) and in 98 patients with CHF (NYHA class 2 to 4) (56 males) during the routine ECG.


**Results:** In multiple backward stepwise regression analysis, aoVWV was related positively to age and negatively to dry AppMM index (males' total *r* = 0.73, females' total *r* = 0.53, both *P* < 0.001). In contrast, DXA‐derived AppMM index uncorrected for ECF excess and body fat were excluded in the stepwise backward regression analysis.


**Conclusions:** DXA‐derived AppMM that is not corrected for ECF excess overestimates AppMM by up to 30% in patients with CHF because of overhydration. In contrast, appendicular muscle mass measured by six‐segment multifrequency impedance corrected for ECF excess can classify sarcopenia with greater precision. The inverse relation between dryAppMM index and aortic volume wave velocity, both derived during the routine 12 channel ECG, reemphasizes the importance of muscularity for vascular health in CHF.


**6-32**



**The association between urine creatinine excretion and bone mineral density in CKD: from the KNOW‐CKD study**



**Young Youl Hyun**
^1^, Ji Min Han^1^, Hyang Kim^1^, Kook‐Hwan Oh^2^, Curie Ahn^2^ and Kyu‐Beck Lee^1^



^1^
*Department of Internal Medicine, School of Medicine, Kangbuk Samsung Hospital, Sungkyunkwan University, Seoul, South Korea;*
^2^
*Department of Internal Medicine, College of Medicine, Seoul National University, Seoul, South Korea*



**Background:** Previous studies have shown that decreased lean body mass or muscle mass is associated with decreased bone mineral density in individuals with preserved renal function. However, this relationship is uncertain in chronic kidney disease (CKD), where more diverse factors such as decreased renal function and disordered mineral metabolism impact on bone health. The aim of this study was to verify the relationship between muscle mass estimated from creatinine excretion and bone mineral density in a large Korean cohort of CKD.


**Methods:** This cross‐sectional study analysed 1740 participants from the KNOW‐CKD cohort. The bone mineral densities of the lumbar spine, total hip, and femur neck were assessed by dual‐energy X‐ray absorptiometry. Muscle mass was estimated from 24 h urine creatinine excretion (UCr). Participants were divided into three groups according to their UCr.


**Results:** The study participants' mean eGFR was 53.7 ± 32.7 mL/min/1.73 m^2^. Among them, 100 (5.8%) participants had osteoporosis. Osteoporosis was more prevalent among the lower UCr groups (12.2%, 4.7%, and 0.3% for the 1st to 3rd tertile, respectively, *P* < 0.001). For each 100 mg/day increase in UCr, BMD increased by 0.07 for lumbar spine, 0.06 for total hip, and 0.04 for femur neck in multivariate linear regression analysis. In multivariate logistic regression, the OR for osteoporosis compared to the 1st tertile was 0.55 (0.33–0.92, *P* = 0.023) in the 2nd tertile and 0.08 (0.02–0.37, *P* = 0.001) for the 3rd tertile.


**Conclusions:** Creatinine excretion was significantly and independently associated with low BMD and osteoporosis in CKD. Future research is warranted to determine if osteoporosis can be prevented through intervention to increase muscle mass.


**6-33**



**Associations between body composition and prognosis of patients admitted because of acute pancreatitis: a retrospective study**



**Mordechai Shimonov**
^1,2^, Zhana Abtomonova^1^, Asnat Groutz^2,3^, Hadar Amir^2,3^ and Eyal Leibovitz^4^



^1^
*Edith Wolfson Medical Center, Holon, Israel;*
^2^
*Sackler Faculty of Medicine, Tel Aviv University, Tel Aviv, Israel;*
^3^
*Lis Maternity and Women's Hospital, Tel Aviv Sourasky Medical Center, Tel Aviv, Israel;*
^4^
*Department of Internal Medicine A, Yoseftal Hospital, Eilat, Israel*



**Background:** We investigated associations of muscle and visceral fat mass with the prognosis of patients hospitalized with acute pancreatitis. Visceral adiposity was shown to be associated with improved prognosis in patients with acute calculous cholecystitis.


**Methods:** This is a retrospective analysis of patients admitted with acute pancreatitis during 2008–2014. Body composition analysis (Sliceomatic, Tomovision, CA) was performed on CT images at the L3 level. Regression analysis was used to examine associations of body composition with 1 year mortality and 1 year readmission rates.


**Results:** A total of 158 patients were included [mean age 63.7 ± 17.4 years, 91 (57.6%) were male]. Fat was the most abundant tissue (408 ± 180 cm^2^ surface area). None of the prognostic factors examined were associated with 1 year mortality. Values below compared to above the medians for muscle mass and visceral fat were associated with higher mean 1 year readmissions: 1.7 versus 1.0, *P* = 0.02 and 1.6 versus 1.1, *P* = 0.09, respectively. Logistic regression analysis showed an association of high visceral fat with reduced 1 year readmission (OR 0.995, 95% CI: 0.991–1.000, *P* = 0.03). Linear regression analysis showed an inverse correlation of visceral fat mass with the number of 1 year readmissions (HR −0.004, 95% CI −0.008–000, *P* = 0.070).


**Conclusions:** Higher amounts of visceral fat and muscle mass were positively associated with lower recurrent hospitalizations in patients admitted with acute pancreatitis. These results support the importance of nutritional rehabilitation in patients after admission due to acute pancreatitis.


**6-34**



**Cut‐off points of phase angle and its association with sarcopenic, frailty, and physical performance in community‐dwelling Mexican older adults**



**Miriam T. López‐Teros**
^1^ and Oscar Rosas‐Carrasco^2^



^1^
*Universidad Iberoamericana, México, City, Mexico;*
^2^
*National Geriatric's Institute, México, City, Mexico*



**Background:** The phase angle (PhA) is the parameter of the electrical bioimpedance (BIA) expresses changes in the amount and quality of soft tissue mass. PhA can be an important tool to evaluate the clinical outcome or to evaluate the progression of the geriatrics syndromes as sarcopenia, frailty, and low physical performance. The lack of reference values of PhA has limited its use in clinical situations and is unknown the relationship between AF and sarcopenia, frailty, and low physical performance.


**Methods:** It is a cohort of community‐dwelling adults from Mexico City; PhA was measured by BIA ®SECA (50 Hz). Muscle mass by dual‐energy X‐ray absorptiometry (DXA) (Hologic Discovery‐WI; Hologic Inc, Bedford‐MA). The appendicular lean mass index ratio (ALMBMI) was calculated dividing the appendicular skeletal muscle mass by the body mass index. A hand strength by dynamometer (JAMAR Hydraulic Hand Dynamometer, Lafayette, IN). In our study, sarcopenia was defined in accordance with the Foundation for the National Institutes of Health (FNIH) criteria, Fried's criteria to frailty, and short battery physical performance (SPPB) to low physical performance.


**Results:** A total of 498 adults of >50 years and older were included. The mean age was 71.1 ± 9.5, the mean of PhA was 4.6 ± 0.70. The cut‐off point of PhA to frailty was ≤4.1, sensitivity = 80.13%, specificity 68.89% LR (+) 2.57, LR (−) 0.28; AUC = 0.83 95% IC (0.8019–0.8686). The cut‐off point of PhA to sarcopenia was ≤4.1, sensitivity = 80.44%, specificity 71.11% LR (+) 2.78, LR (−) 0.27; AUC = 0.84 95% IC (0.8387–0.9142). The cut‐off point of PhA to low physical performance was ≤4.3, sensitivity = 80.0%, specificity 44.91% LR (+) 1.45, LR (−) 0.44; AUC = 0.70 CI 95% (0.6667–0.7484).


**Conclusions:** The association between PhA, sarcopenia, and frailty has a good sensitivity and AUC, both low specificity. Low physical performance has low sensitivity, specificity, and AUC. PhA could be an early and accessible marker to evaluate future changes in body composition (sarcopenia) and frailty in community‐dwelling older adults.


**6-35**



**Myosteatosis is associated with increased oxidized lipid within dendritic cells in patients with colorectal cancer: dendritic cell dysfunctional: the missing key to unlocking the mystery of myosteatosis?**



**Edward T. Pring**
^1,2,3,4^, Lydia R. Durant^2,4^, Alistair Noble^2,4^, Laura E. Gould^2,3,4^, George Malietzis^2,4^, Thanos Athanasiou^2,4^, Phillip Lung^2,3,4^, John T. Jenkins^2,3,4^ and Stella C. Knight^2,4^



^1^
*George Davies Research Fellowship, Leicester, UK;*
^2^
*BiCyCLE Research Group, St. Mark's Hospital, Harrow, UK;*
^3^
*Department of Surgery, St. Mark's Hospital, Harrow, UK;*
^4^
*Imperial College London, London, UK*



**Background:** Myosteatosis is associated with the oncogenic systemic inflammatory response and poor prognosis and outcomes in patients with colorectal cancer (CRC). CT scan analysis can identify these changes in muscle quality. Dendritic cells (DC), the bodies primary antigen presenting cells, orchestrate the cellular immune response. DC have been shown to take up lipid becoming adipocyte‐like. Oxidized lipid within DC is associated with DC dysfunction, interfering with antigen presentation. A significant association between expression of the fat‐scavenger receptor CD36, which drives lipid uptake, on circulating DC and myosteatosis in CRC patients has been demonstrated. We assessed whether the lipid profile of these key immune cells was associated with myosteatosis in CRC.


**Methods:** Peripheral blood mononuclear cells were isolated from whole blood of preoperative CRC patients and labelled with antibodies to identify DC. Cells were permeabilized to examine intracellular total lipid (using Bodipy dye) and oxidized phospholipids (using the monoclonal antibody to E06). Positive staining was determined using fluorescence minus‐one controls. Analysis of preoperative CT scans (SliceOmatic v5.0) identified the presence of myosteatosis using predefined cut‐off values based on the mean muscle attenuation (Hounsfield units) and BMI.


**Results:** Fourteen CRC patients were included in this analysis, median age 64 (male *n* = 11), seven had myosteatosis. In patients with myosteatosis, the frequency of myeloid‐DC (mDC) and plasmacytoid‐DC (pDC) staining positively for EO6 (oxidized lipid) was significantly greater than in patients without myosteatosis, *P* = 0.026 and *P* = 0.017, respectively. There was no significant difference in total lipid content (Bodipy) between groups, mDC *P* = 0.80 and pDC *P* = 0.19.


**Conclusions:** DC dysfunction caused by increased intracellular oxidized lipid is significantly associated with myosteatosis. There is no significant change in DC total lipid uptake in myosteatosis. CD36 could be an immunotherapy target in CRC patients with myosteatosis—preventing oxidized lipid uptake and allowing appropriate presentation of tumour‐specific antigen to the immune downstream effectors.


**6-36**



**Inflammation‐induced skeletal muscle wasting: emerging role of the NLRP3 inflammasome**


Moritz Eggelbusch^1^, Mariana Vázquez‐Cruz^1^, Gerard de Wit^1^, Bart Everts^2^, Richard T. Jaspers^1^ and **Rob C.I. Wüst**
^1^



^1^
*Laboratory for Myology, Department of Human Movement Sciences, Faculty of Behavioral and Movement Sciences, Vrije Universiteit Amsterdam, Amsterdam Movement Sciences, Amsterdam, The Netherlands;*
^2^
*Department of Parasitology, Leiden University Medical Center, University of Leiden, Leiden, The Netherlands*


Moritz Eggelbusch and Mariana Vázquez‐Cruz equally contributed to this article.

Systemic low‐ and high‐grade inflammation in various acute and chronic diseases is associated with loss of skeletal muscle mass and metabolic dysfunction. Complicating factors in understanding molecular mechanism underlying this loss is that muscle wasting is a multifactorial process, and human tissue under well‐controlled wasting conditions is sparse. To mechanistically study how inflammation induces skeletal muscle atrophy and metabolic dysfunction, we differentiated C2C12 myoblasts into myotubes and treated them with lipopolysaccharide (LPS) to induce a low‐grade (10 ng/mL) or high‐grade (100 ng/mL) inflammation. Within 24 h, LPS reduced muscle fiber diameter by 12 ± 9% (10 ng/mL) and 42 ± 6% (100 ng/mL; both *n* = 150, *P* < .001). A higher concentration of LPS (200 ng/mL) severely disrupted normal fiber morphology. IL‐6 concentrations within the supernatant dose‐dependently increased upon LPS treatment. After 72 h, LPS‐treated muscle fibers remained significantly smaller than vehicle‐treated cells.

While the nucleotide‐binding oligomerization domain‐like receptor family pyrin domain containing 3 (NLRP3) inflammasome is an integral component of the innate immune system, its role in the development of muscle wasting in skeletal muscle is poorly understood. NLRP3 and downstream caspase‐1 mRNA gene expression levels were higher in LPS‐treated myofibers after 24 h, but no IL‐1β was detected in supernatant. While the supplementation of an extracellular danger signal, 5 mM ATP, did not result in more muscle wasting, NLPR3, and (pro‐)caspase‐1 protein concentrations were marginally increased. Using immunofluorescence, we observed that this NLRP3 protein had a high colocalization to mitochondria. These data suggest a primed, but not fully activated, NLRP3 inflammasome in skeletal muscle upon LPS treatment and a possible mechanistic link with mitochondrial dysfunction. Current studies are underway to fully understand the role of the NLRP3 inflammasome and metabolic alterations associated with inflammation‐induced skeletal muscle dysfunction. This also allows compounds aimed to alleviate muscle wasting to be tested.


**6-38**



**Fried frailty phenotype and hand grip strength in advanced cancer patients**



**Pia Weinlaender**
^1^, Sara Hadzibegovic^1^, Alessia Lena^1^, Laura‐Carina Lueck^1^, Ruben Evertz^2^, Anne Letsch^3^, Wolfram Doehner^4^, Ulf Landmesser^5^, Stephan von Haehling^2^, Stefan D. Anker^6^ and Markus S. Anker^1^



^1^
*Division of Cardiology and Metabolism, Department of Cardiology, Charité and Berlin Institute of Health Center for Regenerative Therapies (BCRT), DZHK (German Centre for Cardiovascular Research), partner site Berlin and Charité Campus Benjamin Franklin, Berlin, Germany;*
^2^
*Department of Cardiology and Pneumology, Heart Center Göttingen, University of Göttingen Medical Center, Georg‐August‐University, German Center for Cardiovascular Medicine (DZHK), partner site Göttingen, Göttingen, Germany;*
^3^
*Division of Hematology and Oncology, Charité Campus Benjamin Franklin, Berlin, Germany;*
^4^
*Division of Cardiology and Metabolism, Department of Cardiology (CVK), Center for Stroke Research Berlin, Charite Universitätsmedizin, Berlin‐Brandenburg Center for Regenerative Therapies (BCRT), DZHK (German Centre for Cardiovascular Research), partner site Berlin, Berlin, Germany;*
^5^
*Department of Cardiology, Charité‐ Universitätsmedizin Berlin, DZHK (German Centre for Cardiovascular Research), partner site Berlin, Corporate Member of Freie Universität Berlin, Humbolt‐Universität zu Berlin, and Berlin Institute of Health, Berlin, Germany;*
^6^
*Division of Cardiology and Metabolism, Department of Cardiology, Berlin‐Brandenburg Center for Regenerative Therapies (BCRT), DZHK (German Centre for Cardiovascular Research), partner site Berlin, Berlin, Germany*



**Background:** The most used questionnaire for frailty assessment in geriatric patients, the Fried frailty phenotype, has rarely been used in advanced cancer patients.


**Methods:** From November 2017 to July 2019, we prospectively enrolled 178 patients with cancer (61 ± 14 years, 57% men, BMI 25.4 ± 4.7 kg/m^2^, 78% cancer stage ≥3) and 49 healthy controls of similar age and sex. We used the Fried frailty phenotype to assess frailty with 5 domains (each 1 point): shrinking (>10 pounds' unintentional weight loss), weakness (low hand grip strength), poor endurance and energy (self reported), slowness (slow 15‐feet gait speed), and low physical activity level (low energy expenditure). 0 points ≙robust, 1–2 points ≙prefrail, 3–5 points ≙frail.


**Results:** Ninety‐three (52%) patients with cancer were prefrail and 25 (14%) frail. Four (8%) healthy controls were prefrail. In patients with cancer, shrinking, poor endurance and energy, low physical activity level, slowness, and weakness were present in 77 (43%), 30 (17%), 28 (16%), 29 (16%), and 47 (26%), respectively, compared to healthy controls with 2 (4%), 0, 0, 0, 2 (4%). Between the cancer groups, robust, prefrail, and frail, we compared different modes of assessing hand grip strength (average or maximum, dominant or non‐dominant arm, stronger or weaker arm) and found the greatest difference in hand grip strength between the groups when the average of three tries was calculated for the strongest arm (37.0 ± 11.1 kg vs. 29.8 ± 8.9 kg vs. 23.3 ± 8.1 kg, *P* value < 0.0001), respectively. During a median follow‐up time of 10 months (max. 21 months), 35 (20%) cancer patients died. The Fried frailty score was a predictor of survival in univariable (per 1 point, HR 1.38, 95% CI 1.08–1.76, *P* = 0.009) and multivariable analyses (per 1 point, HR 1.29, 95% CI 1.01–1.65, *P* = 0.042), adjusted for tumour type and stage, surgery, and chemotherapy.


**Conclusions:** Ninety‐three (52%) patients with advanced cancer were prefrail and 25 (14%) frail. The Fried frailty score was independently associated with higher mortality.

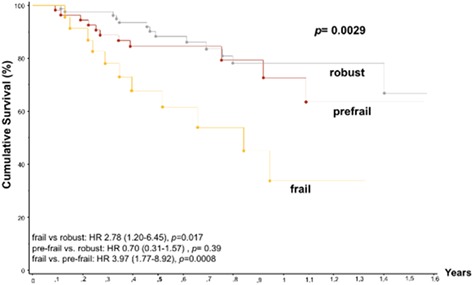




**6-39**



**Cross‐sectional associations between sex hormones, IGF‐1, fat‐free mass, and vitamin D in men aged 50–78 years in the UK EPIC‐Norfolk Study**



**Alisa A. Welch**
^1^, Ryan Janjuha^1^, Diane Bunn^1^, Richard Hayhoe^1^ and Kay Tee Khaw^2^



^1^
*Norwich Medical School, University of East Anglia, Norwich, UK;*
^2^
*Department of Clinical Gerontology, Cambridge University, UK*



**Background:** Fat‐free mass (FFM), an index of skeletal muscle mass, declines with age, contributing to the onset of sarcopenia. The age‐related effects on circulating sex hormones and IGF‐1 also contribute to sarcopenia, as do low concentrations of 25(OH)D. We therefore, investigated associations between circulating concentrations of sex hormones, IGF‐1, and 25(OH)D with FFM, in men aged 50–78 years in the UK EPIC‐Norfolk Study. We further explored whether circulating concentrations of 25(OH)D modified associations between FFM%, sex hormones, and IGF‐1.


**Methods:** Total fat‐free mass (measured using bioelectrical impedance) was calculated as a percentage of body weight (FFM%). Circulating concentrations of testosterone (T), sex hormone‐binding globulin (SHBG), free testosterone (FT), dehydroepiandrosterone sulphate (DHEAS), and IGF‐1, in nmol/L, *N* = 2368, and 25(OH)D (nmol/L, *N* = 1743) were measured. Statistical analyses were performed in STATA (MP V16) with multivariable regression analyses adjusted for the covariates; age, self‐reported physical activity, smoking habit, height, and protein as a percentage of total energy intake (calculated from 7 day food diaries). Concentrations of sex hormones and IGF‐1 were log transformed.


**Results:** Significant correlations were only found between FFM% and T (R 0.14, *P* < 0.001) and SHBG (R 0.25, P < 0.001), FT (R −0.05, *P* = 0.01) which remained after adjustment for covariates in multivariable analysis (T, 0.009% FFM/nmol, *P* < 0.001, and SHBG 0.014% FFM/nmol, *P* < 0.001). The association between 25(OH)D and FFM% was significant in multivariable analysis (0.11% FFM/nmol, *P* < 0.001) but when 25(OH)D was included in the multivariable models, the existing associations between T and SHBG and FFM% did not differ substantially.


**Conclusions:** Although circulating concentrations of testosterone and SHBG are significantly associated with FFM in middle and older aged men, the impact of 25(OH)D on this relationship appears to be small.

## References


(1) 
Janjuha
R
, et al, Welch A, in preparation 2019.



(2) 
Anic
G
, Clin Endo, 85(2):258
2016.10.1111/cen.13062PMC494696626991691



**6-40**



**Use of primary human skeletal muscle cell cultures for the study of chemotherapy‐induced muscle wasting**



**Tatyana Prokhorova**
^1^, Ulrik Frandsen^1^, Per Aagaard^1^, Helle Pappot^2^ and Charlotte Suetta^3^



^1^
*Department of Sports Science and Clinical Biomechanics, University of Southern Denmark, Odense, Denmark;*
^2^
*Department of Oncology, University Hospital of Copenhagen, Rigshospitalet, Copenhagen, Denmark;*
^3^
*Geriatric Research Unit, Bispebjerg‐Frederiksberg and Herlev‐Gentofte Hospital, University of Copenhagen, Copenhagen, Denmark*



**Background:** Muscle wasting during cancer chemotherapy is a known but poorly understood phenomenon that contributes to reduced survival prognosis. In this study, we have established several primary human myoblast cultures to investigate the molecular mechanisms involved in chemotherapy‐induced muscle wasting.


**Methods:** Biopsies from m. vastus lateralis were obtained from healthy donors after informed consent. Satellite cells were derived by a novel explant procedure and myoblasts with myogenic potential subsequently were expanded in growth medium containing 20% fetal bovine serum (FBS). Experiments were performed on differentiated myotubes induced at 80% confluence by complete FBS withdrawal. After initiation of myotube formation (2–3 days) substrate was exchanged to postfusion medium containing 2% FBS. Cisplatin (Pfizer) or Fluorauracil (Hospira Nordic AB) were applied to myotubes for 24 h, and after additional 4–5 days in postfusion medium myotubes were harvested for Western blotting and immunoflourescence analysis.


**Results:** Cells with myogenic potential proliferated and expressed Pax7 in the undifferentiated state. Multinucleated myotubes were found to form in differention medium, expressing myogenin as well as myosin heavy chain (MyHC) isoforms. Exposure to increasing concentrations of cisplatin leads to myotube atrophy concurrent with marked reduction in MyHC expression. As cisplatin concentrations approached levels experienced by chemotherapy patients (5 μg/mL and above), MyHC completely disappeared. Fluorouracil, on another hand, did not seem to affect either phenotype or MyHC expression to any significant extent.


**Conclusions:** In the present study, we have developed and validated a cell based *in vitro* assay to study the physiological effect of chemotherapeutic agents on muscle cells *in vitro,* using biopsy samples from human skeletal muscle. Our findings indicate that not all cancer drugs induce muscle loss to the same extent, cisplatin being markedly more muscle‐toxic than fluorouracil. The present assay system may help to study cellular mechanisms and pathways involved in chemotherapy‐induced muscle wasting. Further, the present approach provides an important possibility to study muscle wasting induced by chemotherapy, allowing to distinguish this from cachexia induced by other factors such as cancer itself or age. Muscle wasting in cancer patients is a major prognostic factor. An improved understanding of the underlying mechanisms is imperative to improve therapy efficiency, to which ends the present *in vitro* assay system may serve as an important tool.


**6-41**



**The influence of aetiology on body composition and muscle strength in male patients with heart failure**


Guilherme Wesley Peixoto da Fonseca^1^, Tania Garfias Macedo^2^, Nicole Ebner^2^, Marcelo Rodrigues dos Santos^1^, Francis Ribeiro de Souza^1^, Liliam Takayama^3^, Rosa Maria Rodrigues Pereira^3^, Stefan D. Anker^2^, Carlos Eduardo Negrão^1,4^, Maria Janieire de Nazaré Nunes Alves^1^ and Stephan von Haehling^2^



^1^
*Heart Institute (InCor), University of São Paulo Medical School, São Paulo, Brazil;*
^2^
*Department of Cardiology and Pneumology, University Medicine Göttingen (UMG), Göttingen, Germany;*
^3^
*Bone Metabolism Laboratory, Rheumatology Division, University of São Paulo Medical School, São Paulo, Brazil;*
^4^
*School of Physical Education and Sports, University of São Paulo, São Paulo, Brazil*



**Background:** Ischaemic patients with heart failure (HF) have worse outcomes in comparison to their nonischaemic counterparts. Moreover, patients with HF and Chagas' disease have also shown to present a higher mortality rate compared to other aetiologies, including ischaemic patients. However, the impact of aetiology on body composition and muscle strength in patients with HF is still unknown. This aims to evaluate the influence of aetiology on body composition and muscle strength in patients with HF from dilated, ischaemic, and chagasic origin in two distinct cohort studies [Brazil (Testo‐HF) and Germany (SICA‐HF)].


**Methods:** We enrolled 64 male patients with HF matched by body mass index (BMI), 20 dilated and 22 ischaemic patients from SICA‐HF (Göttingen, Germany), and 22 chagasic patients from TESTO‐HF (São Paulo, Brazil). All patients were in New York Heart Association functional class II–III (NYHA) with left ventricular ejection fraction (LVEF) ≤40%. Dual‐energy X‐ray absorptiometry was used to assess lean body mass (LBM) and fat mass (FM). Muscle strength was measured by handgrip strength. All patients underwent a maximal cardiopulmonary exercise testing on cycle ergometer (Brazil) or treadmill (Germany). Venous occlusion plethysmography was used to measure forearm blood flow (FBF). Blood samples were also drawn in the morning after an overnight fasting.


**Results:** Patients with dilated HF had higher FM (27.6 ± 9.4 vs. 19.3 ± 8.0 vs. 16.3 ± 8.1 kg; *P* < 0.05) and higher peak absolute VO_2_ (1.50 ± 0.45 vs. 1.15 ± 0.36 vs. 1.17 ± 0.36 L/min; *P* < 0.05) than ischaemic and chagasic patients, respectively. Chagasic patients showed a trend towards decreased LBM when compared with ischaemic patients (48.3 ± 7.6 vs. 54.2 ± 6.3 kg; *P* = 0.094). Moreover, patients with Chagas' disease showed lower handgrip strength (27 ± 8 vs. 37 ± 11 vs. 36 ± 14 kg; *P* < 0.05) and FBF (1.84 ± 0.54 vs. 2.75 ± 0.76 vs. 3.42 ± 1.21; *P* < 0.01) compared with ischaemic and dilated patients, respectively. In addition, FBF correlated positively with handgrip strength (*r* = 0.36; *P* = 0.004) and LBM (*r* = 0.31; *P* = 0.012). In a logistic regression, haemoglobin (hazard ratio, 1.560; 95% confidence interval, 1.043–2.177; *P* = 0.029) and appendicular skeletal muscle mass (hazard ratio, 1.179; 95% confidence interval, 1.011–1.374; *P* = 0.035) were independently associated with peak VO_2_ (median value <1.15) adjusted for age, LVEF, NYHA, creatinine, and FBF. There was no difference between the three groups for laboratory characteristics.


**Conclusions:** Muscle strength showed to be reduced in male patients with HF and Chagas' disease. In addition, reductions in muscle blood flow were also correlated with impaired function and lean body mass, whereas appendicular skeletal muscle mass was independently associated with reduced functional capacity in these patients.


**6-42**



**A novel link between the ubiquitin proteasome system and mitochondrial function to control muscle metabolism**


Vanina Romanello


*Venetian Institute of Molecular Medicine, Department of Biomedical Sciences, Myology Center, University of Padova, Padova, Italy*


Skeletal muscle metabolism of proteins, carbohydrates, and lipids is critical for systemic energy homeostasis. Muscle loss and metabolic alterations in obesity, diabetes, ageing sarcopenia, and cancer cachexia reduce the quality of life, increasing mortality. In these catabolic conditions, a failure in mitochondrial quality control pathways is deleterious for the maintenance of muscle function. However, how mitochondrial dysregulation in muscles contributes to these metabolic disorders is not clear. To address this issue, here we dissect the role of the novel circadian muscle‐specific E3 ubiquitin ligase Asb2b. Asb2b is so far, the only E3 ubiquitin ligase sufficient to cause muscle atrophy when overexpressed in muscles. However, the mechanisms involved and which are its specific substrates are unexplored issues. To unravel its cellular function, we generated muscle‐specific Asb2b knockout mice. Our data show fiber‐type switching, oxidative stress, and several abnormalities in the mitochondrial distribution, turnover, and function in Asb2b‐null mice. Under catabolic conditions, fibers lacking Asb2b cannot cope with energy stress inducing alterations in the metabolism of glucose, suggesting that Asb2b is required for fiber function and integrity during an energy crisis. In addition, the expression levels of Asb2b are induced in dietinduced obesity, ageing sarcopenia, and cancer cachexia. Thus, Asb2b is central in mitochondrial homeostasis which impacts on both muscle mass and metabolism.


**7-09**



**A nutritional supplementation with leucine improved the functional activity of Walker 256 tumour‐bearing Wistar rats**


Lucas Oroy^1,2^, **Laís Rosa Viana**
^1^, Rogerio William Dos Santos^1^, André Schwambach Vieira^3^ and Maria Cristina Cintra Gomes‐Marcondes^1^



^1^
*Laboratory of Nutrition and Cancer, Department of Structural and Functional Biology, Biology Institute, University of Campinas (UNICAMP), Campinas, Brazil;*
^2^
*Polytech Angers, Biology and Health Systems Department, University of Angers, Angers, France;*
^3^
*Laboratory of Electrophysiology, Neurobiology and Behaviour – LENC, Department of Structural and Functional Biology, Biology Institute, University of Campinas (UNICAMP), Campinas, Brazil*


Lucas Oroy, Laís Rosa Viana, and Rogerio William Dos Santos contributed equally to this work


**Background:** Cancer cachexia is characterized mainly by anorexia and involuntary weight loss, with intense muscle and fat mass waste. These symptoms jeopardize patient's quality of life, reducing survival during treatment. A leucine‐rich diet has been shown to increase muscle anabolism and minimize the catabolism. This study aimed to evaluate the effects of a leucine‐rich diet in the functional activity in an experimental model of cachexia.


**Methods:** Wistar adult rats were distributed into four groups: control (C, *n* = 5) and Walker 256 tumour‐bearing (W, *n* = 7), both groups fed a control diet (18% protein) and leucine (L, *n* = 6), and leucine tumour‐bearing (LW, n = 7) groups fed a leucine‐rich diet (18% protein +3% leucine). All rats were monitored, weighed, and the food intake measured three times/week. Their functional activity was assessed, during the night time, analysing the distance covered (cm), the average velocity (cm/s) and the time spent in movement (s) using the software *EthoVisionXT12*. After 21 days of tumour evolution and diet administration, all groups were euthanized, and their serum was collected to measure total protein, albumin, and glucose concentration. Moreover, tibialis anterior muscle and perirenal adipose tissue were collected, weighted, and then normalized by the tibia length.


**Results:** The tumour growth induced cachexia in rats, resulting in weight loss, decreased food intake, muscle and fat mass loss, and impaired the functional activity. Serum biochemical analyses were reduced in both tumour‐bearing groups (*P* < 0.05). However, as a benefit of leucine‐rich diet, LW group maintained the fat mass (LW = C; W < C*, P* = 0.0375), lose less body weight (LW > W, *P* = 0.0252), and showed a better physical functional activity (speed and time moving, LW *>* W, *P* < 0.05).


**Conclusions:** Rats fed a leucine‐rich diet have a better functional activity (faster, more mobile, and resilient) than rats with the same health status under a conventional diet.


**7-10**



**Effects of garlic extract on cancer‐induced muscle wasting**


Ji‐Won Heo^1^, A‐Reum Kim^1^, Hyejin Lee^2^, Jae‐Ha Ryu^3^ and **Sung‐Eun Kim**
^1^



^1^
*Department of Food and Nutrition, Sookmyung Women's University, Seoul, South Korea;*
^2^
*Research Institute of Pharmaceutical Sciences, Sookmyung Women's University, Seoul, South Korea;*
^3^
*Research Center for Cell Fate Control and College of Pharmacy, Sookmyung Women's University, Seoul, South Korea*



**Background:** Cancer cachexia is characterized by weight loss mainly due to ongoing loss of skeletal muscle mass, leading to progressive functional impairment. Skeletal muscle atrophy is considered as the most important factor of cancer cachexia and is ineffectively reversed by nutritional support. Thus, in this study, we investigated whether garlic extract (GE) would alleviate muscle atrophy in a mouse model of colon cancer cachexia in order to provide evidence for candidates to prevent and/or treat cancer cachexia.


**Methods:** After inducing a xenograft model with CT26 cells in BALB/c mice, animals were treated with 0 (Tumor Control, TC), 5 mg/kg (GE5), 10 mg/kg (GE10) for a week. Muscle tissue weights, and the cross‐sectional area were measured. The mRNA expression of markers related with inflammation and protein degradation was determined by RT‐qPCR. Statistical analysis was conducted by one‐way ANOVA followed by Duncan's post hoc test using SAS software version 9.4.


**Results:** GE administration effectively suppressed total muscle weight loss (*P* = 0.01) and muscle fiber atrophy (*P* < 0.0001). In the mice treated with GE, the mRNA expression of E3 ubiquitin ligases was significantly decreased compared with TC group. Similarly, the levels of pro‐inflammatory cytokines and genes associated with JAK/STAT3 signalling pathway were significantly reduced in response to GE supplementation.


**Conclusions:** Our data indicate that GE improves skeletal muscle atrophy induced by cancer cachexia through downregulating systemic inflammatory response and expression of muscle protein catabolic markers. Therefore, these findings suggest that GE has beneficial effects on the prevention and/or treatment of cancer cachexia.

This research was supported by the Basic Science Research Program through the National Research Foundation of Korea (NRF) funded by the Ministry of Education (NRF‐2016R1A6A3A11934151 to S‐EK)


**7-11**



**Prevalence of malnutrition‐sarcopenia syndrome in Mexican older adults living in nursing homes**



**Maria Consuelo Velazquez‐Alva**
^1^, Maria Esther Irigoyen‐Camacho^1^, Marco Antonio Zepeda‐Zepeda^1^, Irina Lazarevich^1^, Fernanda Cabrer‐Rosales^1^ and Isabel Arrieta‐Cruz^2^



^1^
*Department of Health Care, Metropolitan Autonomous University‐Xochimilco Unit, Mexico City, Mexico;*
^2^
*Department of Basic Research, National Institute of Geriatrics, Mexico City, Mexico*



**Background:** Sarcopenia is a geriatric syndrome involving multiple factors among which malnutrition is present. The coexistence of these clinical conditions has resulted in malnutrition‐sarcopenia syndrome (MSS) whose identification is relevant due to increased risk of adverse clinical outcomes. The objective of this study is to identify the prevalence of malnutrition, sarcopenia, and MSS in a group of elderly living in nursing homes.


**Method:** A cross‐sectional study was performed in old people over 65 years living in public nursing homes in Mexico City. The diagnosis of sarcopenia was obtained using the EWGSOP criteria (2010), gait speed (<0.8 mts/sec), hand grip strength (<30 kg for men and <20 kg for women), and muscle mass (calf circumference <31 cm). Malnutrition was assessed using the Mini Nutritional Assessment (<17 points). The protocol was registered by the Council of the Division of Biological Sciences and Health of Metropolitan Autonomous University Xochimilco campus and accepted by the Ethic Committee in Mexico City. The objectives and procedures of this study were explained to the nursing home residents, and all participants signed a written consent form.


**Results**: A total of 212 institutionalized elders were evaluated. 70.3% were women with an average age of 83.4 ± 80 years old. The average age of men was 79.9 ± 8.8 years old. The prevalence of sarcopenia was of 61.7% in women and 39.7% in men. The prevalence of malnutrition was of 32.2% in females and of 17.5% in males. Nevertheless, the prevalence of MSS was of 14.3% in old men and 29.5% in old women.


**Conclusions:** This study demonstrated a high prevalence of MSS among elderly patients living in nursing homes. There is a need to establish intervention programs with nutritional support to prevent and improve nutritional status as well as muscle mass, strength, and function of the skeletal muscle.


**7-12**



**Investigating the relationship between markers of nutritional status, sarcopenia, and frailty and clinical outcomes in older hospital patients**


Hsin‐Hsaio Tsai^1^, David Smithard^2^, Ian Swaine^3^ and **Adrian Slee**
^1^



^1^
*Division of Medicine, University College London, London, UK;*
^2^
*Queen Elizabeth Hospital, Woolwich, London, UK;*
^3^
*University of Greenwich, Avery Hill Campus, London, UK*



**Background:** Malnutrition, sarcopenia, and frailty are likely to interact with each other and may affect clinical outcomes such as length of hospital stay (LOS) and risk of mortality in older hospitalized patients. These conditions should be identified early to inform medical care provision, especially nutritional care. The Nutritional Screening Tool (NST) and Geriatric Nutritional Risk Index (GNRI) are simple malnutrition screening tools, SARC‐F, for sarcopenia, and the Clinical Frailty Scale (CFS) is a recognized for frailty screening. Certain routine blood markers, namely albumin and C‐reactive protein (CRP), and the CRP/albumin ratio may assist in predicting an adverse clinical outcome. This study aimed to investigate the prevalence of malnutrition, sarcopenia, and frailty in acutely hospitalized older patients and relationships between clinical outcomes (LOS and in‐hospital mortality).


**Methods:** A retrospective clinical audit of 139 hospital medical records of older patients aged over 80 years. Data regarding NST, CFS, SARC‐F screening, routine blood markers (urea, albumin, CRP, CRP/Alb ratio), LOS in hospital, and in‐hospital mortality were recoded into an excel database.


**Results:** Over 30% patients were malnourished; 60–65% patients were sarcopenic or frail. NST, SARC‐F, CFS, CRP, and CRP/Alb levels were significantly different between alive and deceased groups (*P* < 0.05). The CFS and CRP were the strongest predictors of death (CFS = CRP > SARC‐F > NST > GNRI). Only GNRI (*r* = 0.45) and NST (*r* = 0.38) demonstrated a fair negative correlation with LOS, while other markers had relatively poor relationships (*r* = 0.2). There was a significant overlap between patients with sarcopenia, frailty, and malnutrition irrespective of whether the NST or GNRI was used for malnutrition screening.


**Conclusions:** The results support the hypothesis that sarcopenia and frailty coexist with malnutrition, though the exact relationship has not been demonstrated. CFS, CRP, and CRP/Albumin ratio are useful indicators of clinical outcomes in older patients.


**7-13**



**Obesity as risk factor for frailty in older Chileans**



**Cecilia Albala** and Lydia Lera and Bárbara Angel and Carlos Marquez and Rodrigo Saguez and Mario Moya


*INTA, Universidad de Chile, Macul, Chile*



**Background:** Although obesity has been shown as a protective factor for survival in older Chileans, this does not necessarily mean that obese people are ageing healthy. The objective of this study is to determine the risk of frailty associated with obesity in older Chileans.


**Methods:** Follow‐up of ALEXANDROS cohorts designed to study disability associated with obesity in community‐dwelling people 60 years and older living in Santiago/Chile. From 1416 participants (67.7% women, mean age 72 years ± 6.7) with baseline anthropometric measurements and those measures needed for the diagnosis of frailty using the Fried's phenotype criteria (unintentional weight loss of ≥5 kg in the previous 6 months, fatigue/exhaustion, walking speed <0.8 m/sec, difficulty walking, weak handgrip strength women ≤15 kg; men ≤27 kg), 711 subjects were free of frailty at baseline. From them, we were able to follow 463 (median follow‐up 5.3 years). At baseline, the subjects were classified as obese or nonobese using WHO standards of BMI. χ^2^ test and logistic models were used for the analysis.


**Results:** The prevalence of obesity at baseline (BMI ≥ 30) was 31.7%. After 3103 person years of follow‐up, 125 new cases of frailty were identified. The incidence of frailty in the total sample was 4.0/100 person years (obese 4.3/100 person years; nonobese 3.75/100 person years). After age, sex, and lean mass adjusted regression analysis, the RR of frailty in obese people was RR = 3.97 (95% CI 1.20–9.51).


**Conclusions:** Incidence of frailty is almost fourfold in obese subjects when compared with nonobese, putting them at high risk of adverse effects on health as functional limitations and disability.


**Funding:** Fondecyt Grant 1130947.


**7-15**



**Malnutrition is still a big problem in oncology clinics: results form 405 patients**


Beatriz Bartissol^1^, Pedro Miguel Neves^3^ and **Paula Ravasco**
^2,3^



^1^
*Hospital de Vila Franca de Xira, Unidade de Dietética e Nutrição, Vila Franca de Xira, Portugal;*
^2^
*Hospital Universitário de Santa Maria, Universidade de Lisboa, Lisbon, Portugal;*
^3^
*Centro de Investigação Interdisciplinar em Saúde da Universidade Católica Portuguesa, Lisbon, Portugal*



**Background:** Weight loss/nutritional deterioration are common features in cancer patients under active treatments. To understand whether or not their incidence is changing during treatments is of major importance, given the theoretical increased awareness in nutrition in clinical practice. Thus, this study aimed to analyse the impact of cancer treatments on body mass index (BMI) and weight changes throughout the treatments.


**Methods:** Epidemiological, observational, retrospective study that included 405 patients with various types of solid tumours, submitted to chemotherapy as outpatients in Vila Franca de Xira Hospital between 2014 and 2017. Age, gender, diagnosis (location and stage), height (m), and weight (kg) at the beginning and end of the treatments was obtained from records, and BMI was calculated. Differences in weight and BMI at the beginning and end of the treatments were evaluated, as well as differences between the qualitative and quantitative variables considering cancer location and stage.


**Results:** A weight loss of 1.1% was observed in all patients (*P* < 0.001), with 39% losing more than 2.4%, as well as a reduction of 0.3 kg/m^2^ in BMI (*P* < 0.001). These results were more relevant for upper gastrointestinal cancer that showed a weight loss of 4.1% (*P* < 0.001) and BMI reduction of 0.9 kg/m^2^ (*P* < 0,001) and also for stage IV disease that lost 1.9% of weight (*P* < 0.01) and had a BMI reduction of 0.5 kg/m^2^ (*P* < 0.01).


**Conclusions:** Despite clear advances in cancer treatment and nutritional therapy in the last decade, the prevalence of nonintentional weight loss associated with cancer and/or treatments remains alarmingly high. Patients submitted to chemotherapy, especially those with cancer of the upper GI tract and/or advanced disease stage have significant weight loss and deterioration of BMI throughout treatments, indicating a clear need for urgent and consistent nutritional intervention. The present results from a 3 year period might translate a still ineffective nutritional intervention in cancer care.


**7-16**



**The prevalence of sarcopenia and malnutrition in a group of oncologic patient**



**Enza Speranza** and Rosa Sammarco and Delia Morlino and Olivia Di Vincenzo and Marialaura Santopaolo and Lidia Santarpia and Maurizio Marra and Fabrizio Pasanisi


*Internal Medicine and Clinical Nutrition Unit, Department of Clinical Medicine and Surgery, Federico II University Hospital, Naples, Italy*



**Background**: Evaluation of nutritional status is a central point of oncologic patient's approach. Therefore, nutritional assessment to identify the risk of malnutrition or malnourishment should be performed in these patients to obtain an early nutritional‐metabolic evaluation. This study aims to evaluate nutritional status, body composition, and sarcopenia in a group of oncologic patients candidate to parenteral nutrition (PN).


**Methods:** This cross‐sectional study included 54 patients: 33 females (57.6 ± 8.5 years; 55.9 ± 16.7 kg; 22.2 ± 6.4 kg/m^2^) and 21 male (66.4 ± 11.7 years; 68.9 ± 19.2 kg; 23.8 ± 4.7 kg/m^2^) with a primary or secondary neoplasia admitted to Medicine and Surgery wards of the Federico II University Hospital. All patients were evaluated for risk of malnutrition using BMI < 18,5 kg/m^2^, the new ESPEN criteria (FFM corrected for height: FFMI, fat‐free mass index), and the presence of sarcopenia with SM (derived from Janssen's equation) corrected for height (SMI).


**Results:** Body composition was estimated by bioimpedance (Females: FFM 41.3 ± 8.7 kg; FAT 14.7 ± 11.3 kg; FAT 24.0 ± 12.7%; Males: FFM 55.3 ± 11.7 kg; FAT 13.6 ± 15.1 kg; FAT 17.2 ± 17.4%). The prevalence of malnutrition was
with BMI < 18.5 kg/m^2^ was 33.3% in female and 4.8% in male,with low FFMI, was 27.7%; also, if we consider the combined finding of unintentional weight loss could be >10% of habitual weight combined with a low fat‐free mass index FFMI, the prevalence was 6.1%.Eleven women (33.3%) and four male (19%) showed severe sarcopenia with a low SMI.
**Conclusions:** Using ESPEN definition, malnutrition was found more frequently in women with both options. The skeletal muscle mass' loss was significant, and it is reported with a high risk of postsurgical complications, chemotherapy toxicity, and mortality. For these reasons, the evaluation of nutritional status is very important for the prevention of malnutrition, because if not treated, survival of oncologic patients could be worsened.


**7-17**



**The relationship between selenium deficiency and cardiovascular diseases in haemodialysis patients**



**So Mi Kim**
^1^, Hwa young Lee^2^, Ji Hyun Jeon^3^, Eunkyoung Lee^1^ and Jong Tae Cho^1^



^1^
*Department of Nephrology, Dankook Univeristy Hospital, Dankook University, College of Medicine, Cheonan, South Korea;*
^2^
*Department of Nephrology, Jeju national University hospital, Jeju University, College of Medicine, Jeju, South Korea;*
^3^
*Department of Nephrology, Jansarang Clinic, South Korea*



**Background:** Cardiovascular disease is prevalent and main cause of death in haemodialysis (HD) patients. Selenium is an essential trace element, and it has been known to prevent cardiovascular disease by protecting the of oxidative stress using selenium‐dependent glutathione peroxidases. HD patients are more likely to have selenium deficiency due to dietary restriction, malabsorption, altered metabolism, haemodialysis, itself etc. Therefore, we tried to investigate the effect of selenium deficiency on thyroid hormone and cardiovascular diseases in HD patients.


**Methods:** A total of 80 HD patients was enrolled in this cross‐sectional study. The patients were divided into two groups based on the level of serum selenium: 61 patients were normal level, and 19 patients were selenium deficient. The cardiovascular diseases were evaluated using echocardiography, coronary computed tomography, or coronary angiography.


**Results:** There were no significant differences in baseline characteristics, including age, sex, duration of HD, and Kt/V between the two groups. Although there was no significant difference, the prevalence of ischaemic heart disease showed higher tendency in selenium deficient group than that in nonselenium deficient group (52% vs. 32% *P* = 0.06), and it showed similar results in heart failure (HF) and cardiomyopathy between the two groups (HF: 32% vs. 24%, cardiomyopathy: 16% vs 12%).


**Conclusions:** This study showed the higher tendency of heart disease in HD patients with selenium deficiency. The large sample sized studies are needed.


**7-20**



**Calf circumference and risk of coronary heart disease**



**Chung‐Ching Wang**
^1,2^, Wei‐Liang Chen^1,2^, Hui‐Fang Yang^1,2^ and Tung‐Wei Kao^1,2^



^1^
*Division of Family Medicine, Department of Family and Community Medicine, School of Medicine, National Defense Medical Center, Tri‐Service General Hospital;, Taipei, China;*
^2^
*Division of Geriatric Medicine, Department of Family and Community Medicine, School of Medicine, National Defense Medical Center, Tri‐Service General Hospital, Taipei, China*



**Background:** Emerging evidence showed the calf circumference (CC) was an important index for sarcopenia in the aged population. Because little literature focused on the CC on the cardiometabolic risks, our objective of the study was examining the association between the CC and risk of coronary heart disease.


**Methods:** The data were collected from geriatric physical screenings at the health promotion center in Tri‐Service General Hospital (TSGH) in Taiwan in 2017. This study enrolled the community‐dwelling elderly who aged greater or equal to 65 years old. The trained staff placed the measuring tape around the participant's right calf to acquire the maximal circumference to the nearest 1.0 millimeter in a sitting position. Framingham risk score of coronary heart disease (FRS‐CHD) was calculated.


**Results:** We examined the CC in 1223 participants (540 in men and 683 in women) with quartiles to identify the demographic characteristics. A significantly negative correlation was noted between the FRS‐CHD and the CC in both genders in all models (all *P* < 0.05) (Table 1). Table 2 listed the baseline characteristics of female participants classified by CC quartiles. Relatively younger age, higher BMI, higher uric acid, lower HDL‐C, and higher prevalence of hypertension treated with antihypertensive agents were found with statistical significance in the higher CC quartiles compared with the lowest quartile (*P* < 0.05). The characteristics of male participants divided by CC quartiles with the mean age of 75.60 ± 8.44 years were presented in Table 3. Table 4 exhibited a significantly negative correlation in higher quartiles of CC with FRS‐CHD in both genders compared with the lowest quartile (*P* < 0.001). Additionally, both men and women, the individuals in the higher quartiles of CC seemed to have a lower FRS‐CHD with the significant association (*P* for trend <0.001).


**Conclusions:** Our findings highlighted that there is a negative association between CC and FRS‐CHD in the elderly population. These findings might underscore the importance of recognizing significant CC atrophy or wasting for future cardiovascular event in the clinical practice.


**7-21**



**Evaluation of useful and convenient screening methods for cardiac cachexia in outpatients with heart failure**



**Norio Suzuki**
^1^, Keisuke Kida^2^, Shunichi Doi^3^, Kohei Ashikaga^3^, Hisao Matsuda^1^, Koichi Mizuno^1^, Tomoo Harada^3^ and Yoshihiro J. Akashi^3^



^1^
*Division of Cardiology, Department of Internal Medicine, St. Marianna University Yokohama City Seibu Hospital, Yokohama, Japan;*
^2^
*Department of Pharmacology, St. Marianna University School of Medicine, Kawasaki, Japan;*
^3^
*Division of Cardiology, Department of Internal Medicine, St. Marianna University School of Medicine, Kawasaki, Japan*



**Background:** Cardiac cachexia is a poor prognosis, and diagnosis is important. However, diagnosis of cardiac cachexia for outpatients is not sufficiently performed because it may be time‐consuming. We investigated screening methods useful for the diagnosis of cardiac cachexia in the clinical setting.


**Methods**: Totally 128 outpatients with CHF aged over 65 years old were enrolled. The criteria for evaluating cachexia by Evans were used. Nutritional status was assessed by the Mini Nutritional Assessment Short Form (MNA○R‐SF) and Geriatric Nutritional Risk Index (GNRI). Frailty was assessed by the “Kihon checklist”. We compared and evaluated these assessments and the diagnosis of cachexia.


**Results**: The mean age was 76.0 ± 7.4 years old, and left ventricular ejection fraction was 43.6 ± 17.2%. Of the study patients, 54.7% patients were male, 20.7% patients had ischaemic heart failure, 45.9% patients had MNA○R‐SF score ≤ 11, and 14.4% patients had cardiac cachexia. The 1 year event‐free survival rates were cardiac cachexia group 64.7% and noncardiac cachexia group 88.5% (log‐rank, *P* < 0.01). The multivariate logistic regression analysis suggested that MNA‐SF score {odds ratio (OR), 0.51; 95% confidence interval (CI), 0.36–0.66; *P* < 0.01}, GNRI (OR, 0.86; 95% CI, 0.79–0.92; *P* < 0.01) and Kihon checklist score (OR, 1.35; 95% CI, 1.19–1.59; *P* < 0.01) might be independent predictors for cardiac cachexia in heart failure patients.


**Conclusions**: Evaluation by MNA○R‐SF, GNRI, and Kihon checklist score was simply performed by blood test or questions, which were useful for diagnosing cachexia.


**7-22**



**Nutritional interventions to improve body composition in persons living with HIV: a systematic review of clinical studies**


Marcus Vinicius Lucio dos Santos Quaresma^1,2^, Camila Maria de Melo^3^ and **Sandra Maria Lima Ribeiro**
^1^



^1^
*Public Health School, University of São Paulo, São Paulo, Brazil;*
^2^
*Centro Universitário São Camilo, São Paulo, Brazil;*
^3^
*Federal University of Lavras, Lavras, Brazil*


Persons living with HIV (PLWH), struggle with side effects of the antiretroviral therapy, which includes body composition changes [fat redistribution and fat‐free mass (FFM) reduction], signifying increased risk of developing sarcopenia. Nutrition interventions can be considered important strategies to attenuate this risk. We developed a systematic review identifying nutrition interventions studies aiming to improve body composition in PLWH. We consulted, following Cochrane recommendations, the databases PubMed, Science Direct, Web of Science, Lilacs, Nature, and Google Scholar and hand‐searched grey literature. The search resulted in 853 publications; after exclusion criteria (non‐human studies and reviews), 31 articles remained, 27 of them investigating FFM [15 open‐label or non‐randomized (OL/NR), and 12 double‐blind randomized clinical trials (RCT)]. We found different interventions and different methods of body composition investigation. From OL/NR, only eight studies showed increase in FFM [supplementation or adequacy of protein intake (*n* = 7); low glycemic index diet (*n* = 1)]. The remained seven studies did not show difference in FFM [supplementations with n‐3 fatty acid (*n* = 1), MCT (*n* = 1), protein (*n* = 1), and micronutrient (*n* = 1); nutritional counselling (*n* = 2); dietary modification (*n* = 1)]. From RCT, only three showed significant increase in FFM [supplementations of arginine, glutamine, and HMB (*n* = 1) and protein (*n* = 2)]. The remaining nine studies did not increase in FFM [supplementation with protein (*n* = 3); arginine and n‐3 fatty acid (*n* = 1); arginine, glutamine, and HMB (n = 1); L‐ornithine ‐ alpha‐ketoglutarate (*n* = 1); chromium (*n* = 1); vitamin D (*n* = 1); and chocolate intake (*n* = 1)]. The heterogeneity of the studies turned impossible to run any meta‐analyses. Concluding, more controlled and randomized studies are necessary to find the best nutrition strategies to deal with the risk of sarcopenia in PLWH.


**7-23**



**Malnutrition, treatment interruptions, and adverse events in head and neck cancer undergoing radiotherapy: still a reality?**


Carolina Bento^1^ and **Paula Ravasco**
^2,3^



^1^
*Instituto Português de Oncologia de Coimbra, Unidade de Nutrição, Coimbra, Portugal;*
^2^
*Hospital Universitário de Santa Maria, Universidade de Lisboa, Lisbon, Portugal;*
^3^
*Centro de Investigação Interdisciplinar em Saúde da Universidade Católica Portuguesa, Lisbon, Portugal*



**Background:** Nutrition has been recognized for over three decades as a fundamental long‐term prognostic factor in head and neck cancer patients. Yet still profound impairments are seen in the nutritional status of these patients. To understand how patients are embarking on cancer treatments and whether or not the incidence of weight loss throughout cancer therapy is improving is of major importance. This study aimed to analyse the impact of cancer and cancer treatments on body weight and the relation between weight loss and treatment interruptions, unplanned admissions, and adverse events occurrence.


**Methods:** Age, gender, tumour location and stage, height, and weight at the beginning and end of the treatments were obtained from the records of 139 patients with head and neck cancer submitted to radiotherapy at the Portuguese Oncology Institute of Coimbra between 1 January 2017 and 4 October 2017. Weight loss at the beginning and end of radiotherapy was evaluated, as well as differences between groups considering cancer location, stage, and treatment modality. Treatment interruptions, unplanned admissions, and adverse events were analysed considering the occurrence of critical weight loss.


**Results:** At baseline, critical weight loss was seen in 60% of the patients with a mean weight loss of 7%. During radiotherapy, 31% of the patients had lost >5% of weight; mean weight loss of 3%. Those undergoing intensive chemoradiation group had a mean weight loss of 7% before and 5% during chemoradiation. No statistically significant differences regarding treatment interruptions, hospital admissions, and adverse events between the two groups of weight loss (critical versus not critical) were found.


**Conclusions:** Despite clear advances in cancer treatment and nutritional therapy, the prevalence of nonintentional weight loss associated with cancer remains high. Patients submitted to chemoradiotherapy have significant weight loss before the diagnosis and throughout treatments, indicating a clear need for an early nutritional intervention.


**7-24**



**Effects of sarcopenia and malnutrition on morbidity and mortality in gynecologic cancer surgery: Results of a prospective cohort study in 237 patients**



**Guelhan Inci**
^*^, Kristina Müller, Rolf Richter, Hannah Woopen and Jalid Sehouli


*Charité – University Medicine of Berlin, Campus Virchow Klinikum, Department of Gynecology with center of surgical oncology, European Competence Center for Ovarian Cancer, Berlin, Germany*



^*^shared authorship


**Background:** Decreased nutritional parameters and muscle attenuation have been associated with poor postoperative outcomes in gynecologic cancer patients. The aim of this study is to evaluate the effect of malnutrition and sarcopenia on postoperative complications.


**Methods:** This is a prospective cohort study of 237 patients undergoing gynecologic cancer surgery at a gynecological cancer center from October 2015 through January 2017. Preoperatively we assessed nutritional parameters including albumin, BMI, the Nutritional Risk Score 2002 and weight loss ≥ 10%. Bio Impedance Analysis (BIA) parameters, such as phase angle alpha, ECM/BCM index, fat mass (FM) and fat free mass (FFM) were evaluated. To assess if patients suffer from sarcopenia we calculated a skeletal muscle index, performed hand grip strength and run the timed up and go test (TUG). More than 400 variables were collected including performance status (ECOG), geriatric assessments and quality of life parameters. Surgical complications were graded using validated Clavien‐Dindo criteria. Using ROC analysis and logistic regression, we identified predictive clinical characters for postoperative complications.


**Results:** Out of the 226 enrolled patients 40 (17.3%) experienced a grade ≥ 3b complication. Within 30 days of surgery, mortality rate was 3.8%. In the regression analysis ECOG > 1 (p = 0.003, OR 6.78, 95% CI: 1.88‐24.48) as well as obesity (p = 0.008, OR 6.63, 95% CI: 1.63‐27) emerged as significant predictors of postoperative complications. Moreover complications were predicted by low albumin < 3,6 g/dl (p = 0.032, OR 3.93, 95% CI: 1.13‐13.69), ECM/BCM ratio (BIA) > 1,35 (p = 0.021, OR 4.21, 95% CI: 1.24‐14.30) and FM > 27.5 kg (p = 0.038, OR 3.06, 95% Cl: 1.06‐8.82) to be independent predictors of increased postoperative complications.


**Conclusion:** In patients undergoing gynecological cancer surgery preoperative evaluation of functional and nutritional health status might help to identify high risk patients to reduce the surgery induced morbidity and mortality in gynecological patients.


**8-11**



**Myosin activity triggers muscle growth through a novel mechanotransduction signalling pathway involving Forcin**


Michael Attwaters and Seetharamaiah Attili and Vladimir Snetkov and Massimo Ganassi and Giorgia Bergamin and **Simon M. Hughes**



*Randall Centre for Cell and Molecular Biophysics, School of Basic and Medical Biosciences, King's College London, London, UK*



**Background:** Physical inactivity leads to skeletal muscle atrophy and weakness in the elderly, hospitalized, and diseased patients and contributes to the obesity epidemic.


**Methods:** To investigate how high‐force exercise promotes muscle growth, we developed a rapid *in vivo* muscle growth assay in the zebrafish larva which was used to test the effect of electrical and optogenetic stimulation and drug treatments on muscle growth. Molecular genetic and biochemical studies analysed signal transduction pathways.


**Results:** Reduced growth of inactive muscle was restored by a brief burst of electrically or optogenetically imposed activity. Activity‐dependent growth involved both increase in muscle fibre size and muscle fibre number, indicating an effect on muscle precursor cells. Growth only occurs if myosin hydrolyses ATP and generates force; imposition of electrical activity in the presence of myosin inhibitors that block contraction without affecting muscle calcium transients fails to elicit growth. Mechanistically, activity promotes TORC1 activity, as assayed by S6 phosphorylation, but myosin blockade does not inhibit TORC1, suggesting that force induces muscle growth through a previously unknown pathway. RNAseq revealed that inactivity reduces and transient imposed activity rescues, in a myosin‐dependent manner, expression of Forcin, an enzyme expressed in striated muscle. Addition of a drug that mimics Forcin activity can substitute for activity and promote growth of inactive muscle. Genome editing was used to ablate Forcin function, and results will be presented.


**Conclusions:** Muscle tissue thus senses activity through several signalling pathways, and a novel mechanosensitive pathway dependent on myosin function is required to trigger growth.


**8-12**



**Muscular strength, physical function, and quality of life in community‐dwelling old adults**



**Alfons Ramel**



*The Icelandic Gerontological Research Institute, Reykjavik, Iceland;*
*Faculty of Food Science and Nutrition, University of Iceland, Reykjavik, Iceland*



**Background:** Quality of life (QoL) has been regarded as a critical predictor of successful ageing in gerontological research. The aim of this study was to examine the associations between physical activity, muscle strength, body composition, physical/cognitive function, and disease with quality of life community‐dwelling older adults.


**Methods:** Participants (*N* = 225, 73.7 ± 5.7 years, 58.2% female) from the Reykjavik capital area in Iceland took part in this cross‐sectional study. Socio‐economics, QoL, body composition, muscular strength, timed up and go test (TUG), 6 min walk for distance (6MWD), and disease‐related information were measured. Fasting blood samples were analysed for routine clinical measures.


**Results:** In our subjects, only 19.1% had QoL below the age and gender corrected norm score of 50. A simple comparison between subjects with QoL below 50 vs subjects with a score above 50 indicated that participants with higher QoL had higher physical and cognitive function, higher muscular strength, lower blood glucose, exercised more, and used a lower number of medicines. Differences in education, smoking, alcohol consumption, dietary intake, and gender distribution were not significant. According to age and gender corrected linear models, TUG (*B* = −0.54, *P* = 0.022), number of drugs (*B* = −0.67, *P* = 0.018), and fasting glucose (*B* = −0.96, *P* = 0.025) were the strongest independent correlates of QoL. In the models, insulin/glucose and TUG/6MWD were interchangeable.


**Conclusions:** Physical function, number of drugs, and glucose metabolism are independently related to QoL and represent therefore potentially modifyable targets for future interventions in order to improve QoL in community‐dwelling old adults.


**8-13**



**Five times sit‐to‐stand test and wrist fracture risk in community‐dwelling obese Icelandic adults**



**Alfons Ramel**
^1,2^, Bergthora Baldursdottir^2,3,4^, Susan L. Whitney^5^ and Palmi V. Jonsson^2,3^



^1^
*Faculty of Food Science and Nutrition, University of Iceland, Reykjavik, Iceland;*
^2^
*The Icelandic Gerontological Research Institute, Reykjavik, Iceland;*
^3^
*Faculty of Medicine, University of Iceland, Reykjavik, Iceland;*
^4^
*Department of Physiotherapy, Landspitali, University Hospital of Iceland, Reykjavik, Iceland;*
^5^
*Department of Physical Therapy, University of Pittsburgh, Pittsburgh, PA, USA*



**Background:** The associations between obesity, physical function, falls, and fractures are unclear. The aim was to investigate whether obese adults have poorer sensory function, physical function as well as higher fracture risk than normal weight peers; and whether confounders explain potential differences between body mass index (BMI) categories.


**Methods:** A case‐control study was conducted using 98 wrist fracture cases (50–75 years) and 48 matched controls. Measurements included among others: anthropometrics, sensory function (Semmes–Weinstein monofilaments), physical function [five times sit‐to‐stand test (FTSTS)], questionnaires on previous fractures and fall history, the activities‐specific balance confidence (ABC), and the Dizziness‐Handicap‐Inventory (DHI) scales.


**Results:** Obese participants had lower physical function (FTSTS: +2.8 s, *P* < 0.001), lower tactile sensitivity (monofilaments:+3.4 g, *P* = 0.013), poorer ABC score (14.0, *P* < 0.001), higher DHI score (11.6, *P* < 0.001), and experienced more falls the previous 12 months (+0.8, *P* = 0.006) compared to normal weight subjects. According to crude logistic regression analysis, the hazard ratios for wrist fracture in overweight and obese subjects were 2.7 (*P* = 0.012) and 5.6 (*P* = 0.002), respectively. When FTSTS was included in the statistical model (HR: 1.7, *P* = 0.004), it reduced the hazard ratios of overweight and obesity close to 1. Lifetime fractures (HR: 5.5, *P* < 0.001) and fall history (HR: 4.3, *P* < 0.001) were associated with an increased fracture risk independently from the BMI categories.


**Conclusions:** Obese individuals have poorer physical function, higher risk of falls, and increased wrist fracture risk compared to normal weight peers. Lower physical function is the main driver for the increased risk in obese subjects. Lifetime fractures and fall history are associated with a fracture independently from obesity.


**8-14**



**Muscle mass, physical function, and bone health in Icelandic community‐dwelling old adults living alone**



**Alfons Ramel**



*The Icelandic Gerontological Research Institute, Reykjavik, Iceland;*
*Faculty of Food Science and Nutrition, University of Iceland, Reykjavik, Iceland*



**Background:** Loneliness and living alone have been significant public health concerns among older adults given their association with a wide range of adverse health outcomes. The aim of this study was to examine whether living alone is associated with physical function and bone health in community‐dwelling older adults.


**Methods:** This was a secondary analysis of existing cross‐sectional data of old adults (*N* = 182, 73.7 ± 5.7 years, 58.2% female) from the Reykjavik capital area in Iceland. Information on socio‐economics, health, dietary intake, and physical function was collected. 25‐hydroxy‐vitamin D (25OHD) and bone mineral density (BMD) were measured. Participants were grouped retrospectively into “living alone” and into “in cohabitation”.


**Results:** Of our subjects, 76.4% were in cohabitation, and 23.6% lived alone. Participants who lived alone were older (74.5 ± 5.6 vs. 72.1 ± 5.0, *P* = 0.008) and more often female (74.4 vs. 53.2%, *P* = 0.014), but there were no differences in education, smoking, number of medications, physical activity (PA), muscle mass, or body mass index (BMI). According to age and gender corrected analyses, participants in cohabitation had higher grip strength (6.2 ± 2.4 lb, *P* = 0.011), higher 25OHD (13.1 ± 6.3 nmol/L, *P* = 0.037), and higher BMD (z‐score lumbal: 1.195 ± 0.417, *P* = 0.005; z‐score femur: 0.421 ± 0.219, *P* = 0.054; z‐score total: 0.846 ± 0.290, *P* = 0.004). Statistical correction for PA, BMI, education, and fish oil intake did not change the results.


**Conclusions:** In comparison to old adults who live in cohabitation, Icelandic old adults who live alone have poorer physical function, lower 25OHD, and lower BMD, which increases their risk for wrist or hip fracture. These differences between groups were not explained by physical, dietary, or social confounding variables.


**8-15**



**Muscle mass, physical function, and quality of life in community‐dwelling old adults who adhere to physical activity according to the Nordic Nutrient Recommendations 2012**



**Alfons Ramel**



*The Icelandic Gerontological Research Institute, Reykjavik, Iceland;*
*Faculty of Food Science and Nutrition, University of Iceland, Reykjavik, Iceland*



**Background:** Regular physical activity (PA) is associated with better health outcomes in gerontological research. The Nordic Nutrient Recommendations 2012 advise to engage in >30 min of PA/day. The aim of this study was to (i) characterize physically active community‐dwelling older adults compared to nonphysically active and (ii) to examine the associations between physical activity and muscle strength, body composition, physical/cognitive function, and disease in this group.


**Methods:** Participants (*N* = 225, 73.7 ± 5.7 years, 58.2% female) from the Reykjavik, Iceland took part in this cross‐sectional study. Physical activity, socio‐economics, quality of life (QoL), anthropometrics, strength, timed up and go test (TUG), 6 min walk for distance (6MWD), and disease‐related information were measured.


**Results:** Of our subjects, 57.2% exercised at least 30 min a day (77 ± 47 min/day), 42.8% exercise less than recommended (11 ± 9 min/day, *P* < 0.001). Physically active participants were younger (72.6 ± 5.7 vs. 74.8 ± 5.5 years, *P* = 0.003) and more often female (65.2 vs. 49.5%, *P* = 0.016), but other socio‐economic differences or dietary intake were not significant. According to linear models corrected for age and gender, PA as recommended was associated with lower BMI (*B* = −1.458 kg/m^2^, *P* = 0.029), lower fat mass (*B* = −3.482 kg, *P* = 0.014), lower muscle mass (*B* = −1.674, *P* = 0.022), higher quadriceps strength (*B* = 36.2 N, *P* = 0.010), higher 6MWD (*B* = 41.2 m, *P* = 0.001), lower TUG (*B* = −0.68 s, *P* = 0.002), and higher QoL (*B* = 2.2, *P* = 0.022) but not with any blood chemical variables, cognitive function, or number of drugs.


**Conclusions:** Physical activity was common in this group of community‐dwelling old adults. Female participants were more active, but otherwise, physical activity was independent from socio‐economic variables. Not unexpected, physical activity was related to better body composition and physical function but neither to medication use nor to blood chemical variables. It can be speculated that a more structured/intense exercise programme is needed to improve these parameters.


**8-16**



**90 day continuous activity recording to characterize physical frailty trajectories in older adults: a tool for modelling wax‐and‐waning conditions in real‐world context**


Luca Carlo Feletti^1^, Gianluca Zia^1,2^ and **Susanna Del Signore**
^2^



^1^
*Caretek Srl, Torino, Italy;*
^2^
*BlueCompanion Ltd, London, UK*



**Background**: We had previously shown (Magistro et al., 2018) how to measure the levels of spontaneous mobility of the elderly subject in a nonintrusive way using a connected accelerometer (ADAMO, worn as an electronic wristwatch). The mobility index (MI) was then defined, a synthetic variable quantifying intensity and temporal distribution of physical activity, on medium‐long term. We also estimated and analysed the gait speed, normalized on the first 15 monitored days (Mulasso et al, 2019). After defining an analytic procedure based on both mean speed and activity level distribution, we applied it to a reference data set, namely the European collaborative study “DECI”.


**Methodology:** We selected the observations lasting 90 days or longer. As Adamo device allows to separate information of outdoor/indoor recording, we restricted the analysis on outdoor activity. The “mobility index distribution” was modelled by assigning a weight to each activity level. Then its “variability” was estimated along the observed period, at least 90 days. Next, the “mean speed” and the “activity level variability” trajectories were compared and merged into a third aggregated trajectory, defining a frailty index. The “frailty index trajectory” was estimated for all 90 day or longer observations available in DECI data set.


**Results and conclusions:** We expected that a mean speed declining trajectory would lead to a worsen frail patient status if the variability trajectory increases. This data behaviour was confirmed within the DECI data set. Then it was possible to define a “frailty index trajectory” as the ratio between the mobility index variability and the mean speed trajectories. The results in the DECI data set are promising. The next step is to apply this approach to a different population, e.g., sarcopenic oncologic patients who underwent multicomponent regimens with the aim of using the frailty trajectory to model the comprehensive longitudinal effect of treatment, in a real‐world context.
